# Branched late-steps of the cytosolic iron-sulphur cluster assembly machinery of *Trypanosoma brucei*

**DOI:** 10.1371/journal.ppat.1007326

**Published:** 2018-10-22

**Authors:** Maiko Luis Tonini, Priscila Peña-Diaz, Alexander C. Haindrich, Somsuvro Basu, Eva Kriegová, Antonio J. Pierik, Roland Lill, Stuart A. MacNeill, Terry K. Smith, Julius Lukeš

**Affiliations:** 1 Biomedical Sciences Research Complex (BSRC), University of St Andrews, St Andrews, Fife, United Kingdom; 2 Biology Centre, Institute of Parasitology, Czech Academy of Sciences, České Budějovice (Budweis), Czech Republic; 3 Faculty of Sciences, University of South Bohemia, České Budějovice (Budweis), Czech Republic; 4 Institut für Zytobiologie, Philipps-Universität Marburg, Marburg, Germany; 5 Faculty of Chemistry–Biochemistry, University of Kaiserslautern, Kaiserslautern, Germany; 6 LOEWE Zentrum für synthetische Mikrobiologie, Marburg, Germany; University of Texas Medical Branch, UNITED STATES

## Abstract

Fe-S clusters are ubiquitous cofactors of proteins involved in a variety of essential cellular processes. The biogenesis of Fe-S clusters in the cytosol and their insertion into proteins is accomplished through the cytosolic iron-sulphur protein assembly (CIA) machinery. The early- and middle-acting modules of the CIA pathway concerned with the assembly and trafficking of Fe-S clusters have been previously characterised in the parasitic protist *Trypanosoma brucei*. In this study, we applied proteomic and genetic approaches to gain insights into the network of protein-protein interactions of the late-acting CIA targeting complex in *T*. *brucei*. All components of the canonical CIA machinery are present in *T*. *brucei* including, as in humans, two distinct CIA2 homologues *Tb*CIA2A and *Tb*CIA2B. These two proteins are found interacting with *Tb*CIA1, yet the interaction is mutually exclusive, as determined by mass spectrometry. Ablation of most of the components of the CIA targeting complex by RNAi led to impaired cell growth *in vitro*, with the exception of *Tb*CIA2A in procyclic form (PCF) trypanosomes. Depletion of the CIA-targeting complex was accompanied by reduced levels of protein-bound cytosolic iron and decreased activity of an Fe-S dependent enzyme in PCF trypanosomes. We demonstrate that the C-terminal domain of *Tb*MMS19 acts as a docking site for *Tb*CIA2B and *Tb*CIA1, forming a trimeric complex that also interacts with target Fe-S apo-proteins and the middle-acting CIA component *Tb*NAR1.

## Author summary

Cytosolic and nuclear proteins containing iron-sulphur clusters (Fe-S) are essential for the survival of every extant eukaryotic cell. The biogenesis of Fe-S clusters and their insertion into proteins is accomplished through the cytosolic iron-sulphur protein assembly (CIA) machinery. Recently, the CIA factors that generate cytosolic Fe-S clusters were characterised in *T*. *brucei*, a unicellular parasite that causes diseases in humans and animals. However, an outstanding question in this organism is the way by which the CIA machinery directs and inserts newly formed Fe-S clusters into proteins. We found that the *T*. *brucei* proteins *Tb*CIA2B and *Tb*CIA1 assemble at a region of the C-terminal domain of a third protein, *Tb*MMS19, to form a complex labelled the CIA targeting complex (CTC). The CTC interacts with *Tb*NAR1 and with Fe-S proteins, meaning that the complex assists in the transfer of Fe-S clusters from the upstream members of the pathway into target Fe-S proteins. *T*. *brucei* cells depleted of CTC had decreased levels of protein-bound cytosolic iron, and lower activities of cytosolic aconitase, an enzyme that depends upon Fe-S clusters to function.

## Introduction

Iron-sulphur (Fe-S) clusters are simple and versatile cofactors involved in a plethora of cellular processes from bacteria to humans and theorised to have formed the ancient surfaces upon which prebiotic chemical reactions took place, laying the ground for the origin of life itself [1,2]. Biogenesis of Fe-S clusters and their subsequent incorporation into polypeptide chains are intricate processes involving dedicated compartmentalised pathways that comprise dozens of proteins [3,4]. At least three such pathways are conserved in eukaryotes, namely the cytosolic Fe-S protein assembly (CIA) machinery, the mitochondrial Fe-S cluster assembly (ISC) system and the plastidial sulphur mobilisation (SUF) system [4–6].

A cytosolic pathway for maturation of Fe-S proteins was first described in the early 2000’s when a genetic screen aimed at the reconstitution of the [4Fe-4S] cluster on human IRP1, also known as cytosolic aconitase, identified the cytosolic P-loop NTPase Cfd1 as essential for the maturation of IRP1 and other cytosolic, but not mitochondrial Fe-S proteins [7]. Since then, at least eight additional proteins (nine in yeast) have been associated with the CIA machinery, which has been implicated in the maturation of a growing list of cytosolic and nuclear Fe-S proteins [4].

The biogenesis of Fe-S proteins can be conveniently simplified in two discrete yet concerted steps: one for assembly of the clusters into a protein scaffold and another for their trafficking/insertion into client proteins. Functional studies have shown that the CIA machinery is highly conserved from yeast to man, and is organised into several sub-complexes that support different stages of the process [8], allowing the components of this pathway to be grouped in a modular fashion as follows: (i) an early-acting module encompassing proteins of the electron transfer chain Tah18 and Dre2 [9], and a heterotetrameric protein scaffold formed by Cfd1 and Nbp35, in which [4Fe-4S] clusters are initially assembled [10,11]; (ii) a middle-acting module, represented by Nar1 [11, 12] and concerned with the transfer and trafficking of the pre-formed Fe-S clusters to (iii) the late-acting or targeting module that facilitates the target-specific insertion of clusters into client proteins [13,14]. In yeast, the CIA targeting complex (CTC) is composed of Mms19, Cia1, and Cia2 [15], while human cells possess two isoforms of Cia2, labelled CIA2A and CIA2B, with the former displaying a notable specificity for the maturation of a subset of client proteins implicated in cellular iron homeostasis, while the latter is involved in canonical Fe-S cluster assembly.

*Trypanosoma* and *Leishmania* species are causative agents of human diseases that threaten hundreds of millions of people mostly in developing countries, as well as of major economically important veterinary diseases [16–19]. *T*. *brucei* is the best-studied member of the supergroup Excavata [20] serving as a model organism due to its genetic tractability [21–24]. The early- and middle-acting modules of the CIA pathway have been previously characterised in this parasite [25], however, the components of the late-acting part had yet to be studied. In addition to this, the Fe-S proteome of this divergent protist remains vastly unexplored, thus providing an excellent opportunity to study these two biological questions.

In this work, we demonstrate that the late-acting module of the CIA machinery is essential for the survival of this parasite *in vitro*, but not *in vivo*. *Tb*CIA2B and *Tb*CIA1 assemble at the C-terminal domain of *Tb*MMS19 to form the canonical ternary targeting complex. Moreover, in both procyclic (PCF) and bloodstream stages (BSF) of *T*. *brucei*, binary configurations reminiscent of those observed in human cells were also present. Members of the CTC interacted with client Fe-S proteins and *Tb*NAR1, while depletion of CTC components impaired cell growth and led to decreased protein-bound cytosolic iron levels and aconitase activity.

## Results

### Identification of CIA-targeting complex and subcellular localisation

Four proteins, termed *Tb*CIA1 (Tb927.8.3860), *Tb*CIA2A (Tb927.9.10360), *Tb*CIA2B (Tb927.8.720) and *Tb*MMS19 (Tb927.8.3920, Tb927.8.3500), were previously identified in *T*. *brucei* on the basis of their similarity to yeast and human CTC components [26,27]. Only *Tb*CIA1 has been characterized to date [25]. *T*. *brucei* encodes two different MMS19 proteins, sharing 99.6% amino acid identity. As in humans, two genes encoding homologues of yeast Cia2 protein were found in *T*. *brucei*. The phylogenetic position of these proteins, designated *Tb*CIA2A and *Tb*CIA2B, has been analysed elsewhere [28].

**Fig 1:**
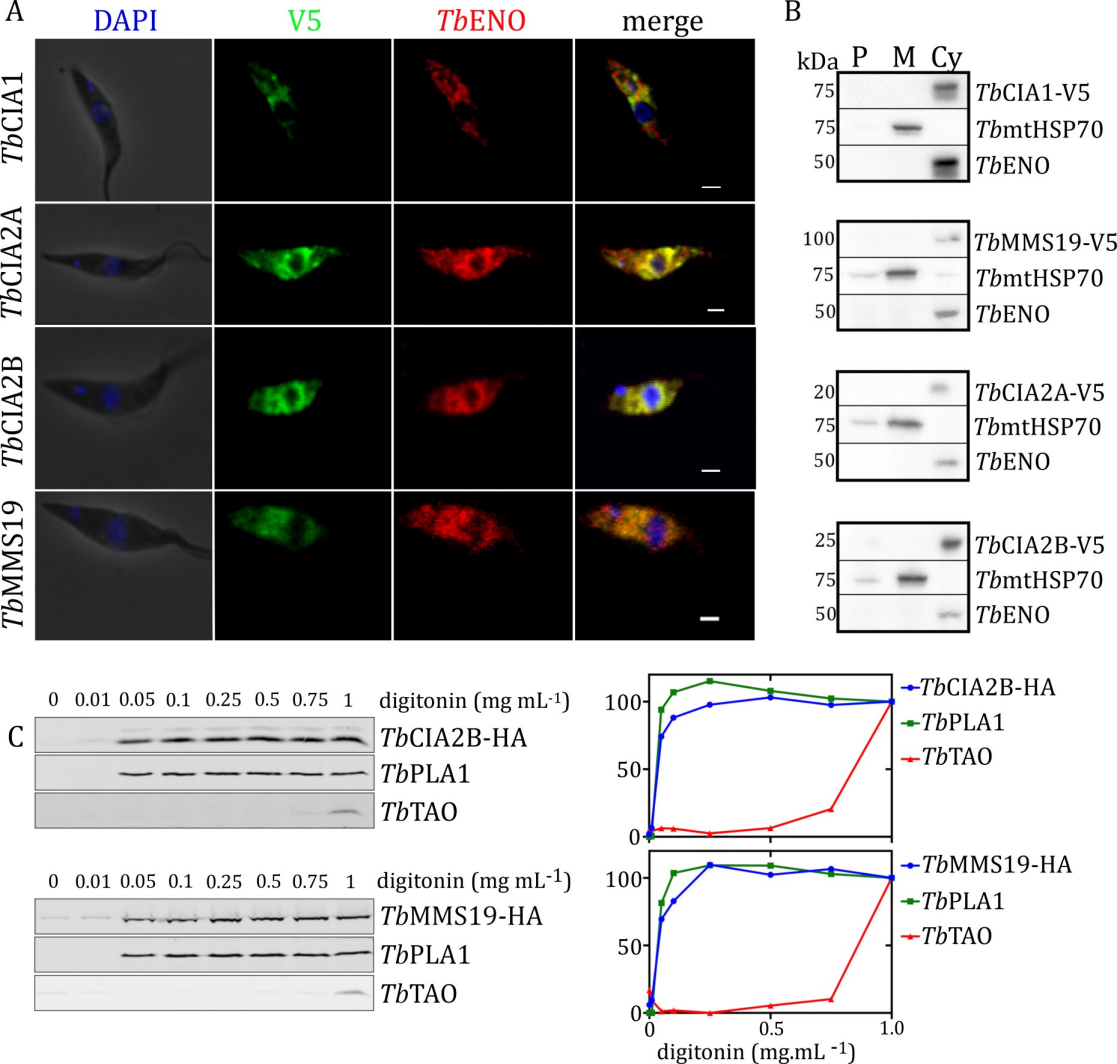
The CIA targeting complex is localised in the cytosol of *T*. *brucei*. **(A)** Confocal microscopy of PCF *T*. *brucei* cells expressing *in-situ* V5-tagged CIA components. Anti-V5 antibody (green) was used to detect the CIA proteins localized throughout the cell body. Enolase (red) was used as a cytosolic marker. DAPI (blue) stained DNA. Scalebar 1 μm. The merge displays co-localization of enolase with the V5-tagged proteins. **(B)** Isolation of mitochondrial fraction with digitonin. PCF trypanosomes were incubated with 0.4% (w/v) digitonin and fractions were separated by centrifugation. V5-tagged targets were visualized with anti-V5 monoclonal antibody. MtHSP70 and enolase were used as mitochondrial and cytosolic markers, respectively. P = pellet; M = mitochondrial fraction; Cyt = cytosolic fraction. All methods indicated that the proteins of the CIA targeting complex are present in the cytosol of PCF *T*. *brucei*.**(C)** Selective permeabilisation of whole PCF *T*. *brucei* cells with digitonin: supernatants of cells incubated with increasing amounts of digitonin were assessed by Western blot. Samples were probed against -HA (CIA components) and organelle markers: *Tb*PLA1 (cytosolic marker); *Tb*TAO (mitochondrial marker). The graphs represent densitometric quantifications of the Western blots for each experiment. Cells permeabilised with the highest concentration of digitonin were used as 100% release control.

We determined the subcellular localisation of *Tb*CIA2A, *Tb*CIA2B, and *Tb*MMS19 by indirect immunofluorescence, crude digitonin fractionation and selective permeabilisation with digitonin. Cell lines expressing *in situ* C-terminally V5- or HA-tagged CIA proteins were produced (see Materials and Methods). Fixed parasites were probed with anti-V5 and anti-enolase antibodies (*Tb*ENO) [29] to detect the fusion proteins and the cytosolic marker, respectively. The co-localisation of all V5-tagged proteins with *Tb*ENO suggests their cytosolic localisation (**Fig 1A**). To further confirm this finding, the subcellular distribution of the CIA pathway components was analysed by a fractionation with digitonin. For this, we incubated the cells with a concentration of digitonin that liberates the cytosol, separating it from the mitochondrial fraction (**Fig 1B**). The signal for all of the CTC components co-localizes with that of the cytosolic marker (Cyt), *Tb*ENO. The mitochondrial marker, *Tb*mtHSP70, is only present in the mitochondrial (M) fraction. The pellet (P) denotes the insoluble fraction after solubilizing the mitochondrial fraction, which exhibits proteins that are membrane-bound, such as part of *Tb*mtHSP70. A parallel corroboration was performed by selective permeabilisation with digitonin. In this experiment, equal numbers of cells were incubated with increasing concentrations of the detergent, causing progressive cell membrane permeabilisation and sequential release of the cytosolic and organellar fractions. *Tb*CIA2B-HA and *Tb*MMS19-HA were co-released with the cytosolic control phospholipase A1 (*Tb*PLA1) [30], while the trypanosome alternative oxidase (*Tb*TAO), which served as a mitochondrial marker [31], was released only at higher detergent concentrations (**Fig 1C**). Taken together, immunofluorescence and detergent-based cell fractionation identified *Tb*CIA1, *Tb*CIA2A, *Tb*CIA2B and *Tb*MMS19 as cytosolic proteins.

### Essentiality and functional analysis of the CIA targeting complex

**Fig 2:**
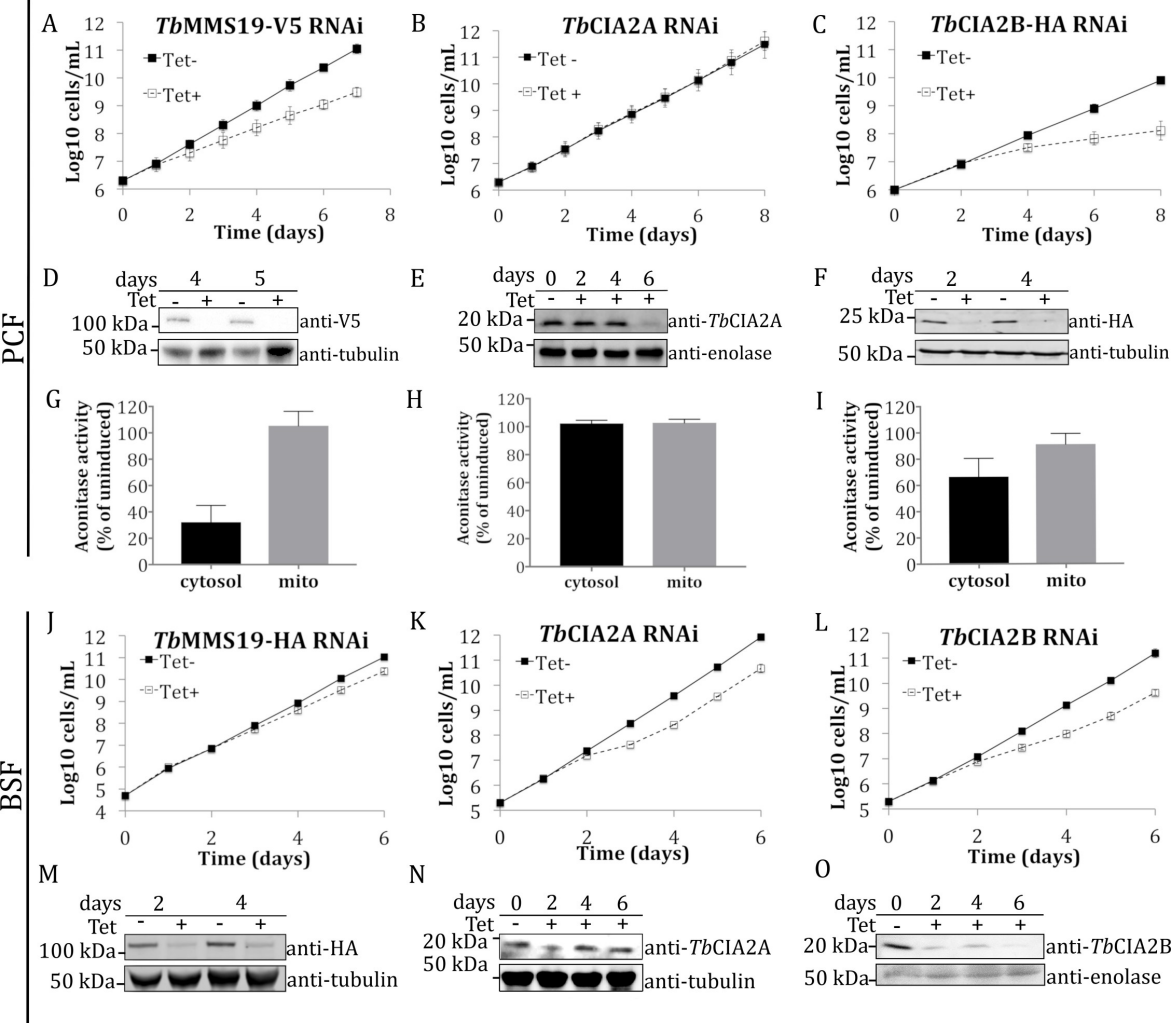
The CIA targeting complex is essential for the cell growth of *T*. *brucei* and activity of cytosolic aconitase. Growth curves of RNAi cell lines in PCF *T*. *brucei* of *TbMMS19*, *TbCIA2A*, and *TbCIA2B* (A-C), induced (Tet +) and uninduced (Tet -) with tetracycline (n = 3 ± SD). Western blots shown under each growth curve were probed with anti-HA, anti-V5 or specific antibodies and were used to assess protein expression before and after RNAi induction (D-F). Anti-tubulin or anti-enolase antibodies were used as loading controls. The activity of the Fe-S dependent enzyme *Tb*ACO was measured in cytosolic and mitochondrial fractions (G-I) of the above-mentioned cell lines (n = 3 ± SD). Growth curves for BSF RNAi cell lines of *Tb*MMS19, *Tb*CIA2A, and *Tb*CIA2B (J-L, n = 3 ± SD). Western blots assessing downregulation of each BSF RNAi cell lines (M-O).

Analysis of the function of the putative CTC members was carried out in uninduced and RNAi-induced PCF and BSF cell lines. The efficiency of the RNAi knockdowns was monitored for up to 8 days in the PCF and 6 days in the BSF and was further assessed by Western blot analysis (**Fig 2A–2F** and **2J–2O**). While the growth rate of *Tb*MMS19 RNAi in the BSF was mostly unaffected upon depletion, the same downregulation in the PCF exhibited considerable growth impairment (**Fig 2A** and **2J**). On the other hand, the BSF *Tb*CIA2A RNAi cell line showed a mild growth phenotype (**Fig 2K**), whereas in PCF this downregulation does not affect the growth rate (**Fig 2B**). Two days after the downregulation of *TbCIA2B*, a decrease in the growth of the PCF was observed (**Fig 2C**), but this effect was less pronounced in the BSF (**Fig 2L**). We have previously shown that depletion of the scaffold proteins *Tb*CFD1 and *Tb*NBP35 caused mild to severe growth impairment in PCF and BSF, but knocking down the expression of individual components upstream of the CTC did not affect the growth rate [25]. However, stringent pairwise knockdowns of the early-acting components of this pathway (e.g. *Tb*TAH18 and *Tb*DRE2) caused marked growth defects [25], suggesting an interaction of CIA factors, which only becomes critical upon simultaneous RNAi knockdown of more than one of them. We sought to employ this phenomenon by silencing the expression of two CTC members simultaneously. However, the observed phenotype of double knockdowns in PCF (*Tb*CIA1-*Tb*CIA2B and *Tb*CIA2A-*Tb*CIA2B) was no more pronounced than the phenotype observed following the depletion of *Tb*CIA2B alone (**S1 Fig**).

To assess the role of the CTC components on the pathogenicity of *T*. *brucei*, we infected mice with BSF RNAi cell lines of *Tb*MMS19 and *Tb*CIA2B. As shown in S2 Fig, these infection experiments suggest that neither protein is essential in BSF, in agreement with the mild *in vitro* growth phenotypes described above, as well as with the initial observation of the double-knockdown cell lines in this life stage, in which at least two components of the pathway had to be ablated in order to obtain a clearer growth phenotype (**S1 Fig**) [25]. We next asked whether depleting the cells of individual CTC members would impact the activity of known Fe-S proteins. Aconitase (*Tb*ACO), a Fe-S enzyme that catalyses the reversible isomerisation of citrate to isocitrate, is encoded by a single gene and has a dual subcellular localisation, being ca. 70% in the cytosol and 30% in the mitochondrion [32,33]. These features qualify it as a suitable surrogate for Fe-S cluster-dependent enzymatic activity in these two cellular compartments [27]. As shown in **Fig 2G** and **2I**, cytosolic *Tb*ACO activity was reduced in 60% and 40%, when *Tb*MMS19 and *Tb*CIA2B were knocked down, respectively, whereas the mitochondrial activity remained unchanged. Furthermore, the depletion of *Tb*CIA2A did not affect aconitase activity in these cellular compartments (**Fig 2H**). Hence, *Tb*MMS19 and *Tb*CIA2B seem to be required for the maturation of this Fe-S protein, providing a functional link between the CTC and the transfer of Fe-S clusters to target proteins.

Several lines of evidence have linked the pathways for Fe-S cluster biogenesis to DNA repair processes in humans, yeast, and plants [13,14,34–37]. Surprisingly, even efficient depletion of the CTC members did not affect the ability of *T*. *brucei* to cope with DNA damage caused by various genotoxic agents as determined by Alamar blue assays, and in some cases the EC_50_ was in fact higher for the CIA-depleted parasites (**S1 Table**).

**Fig 3:**
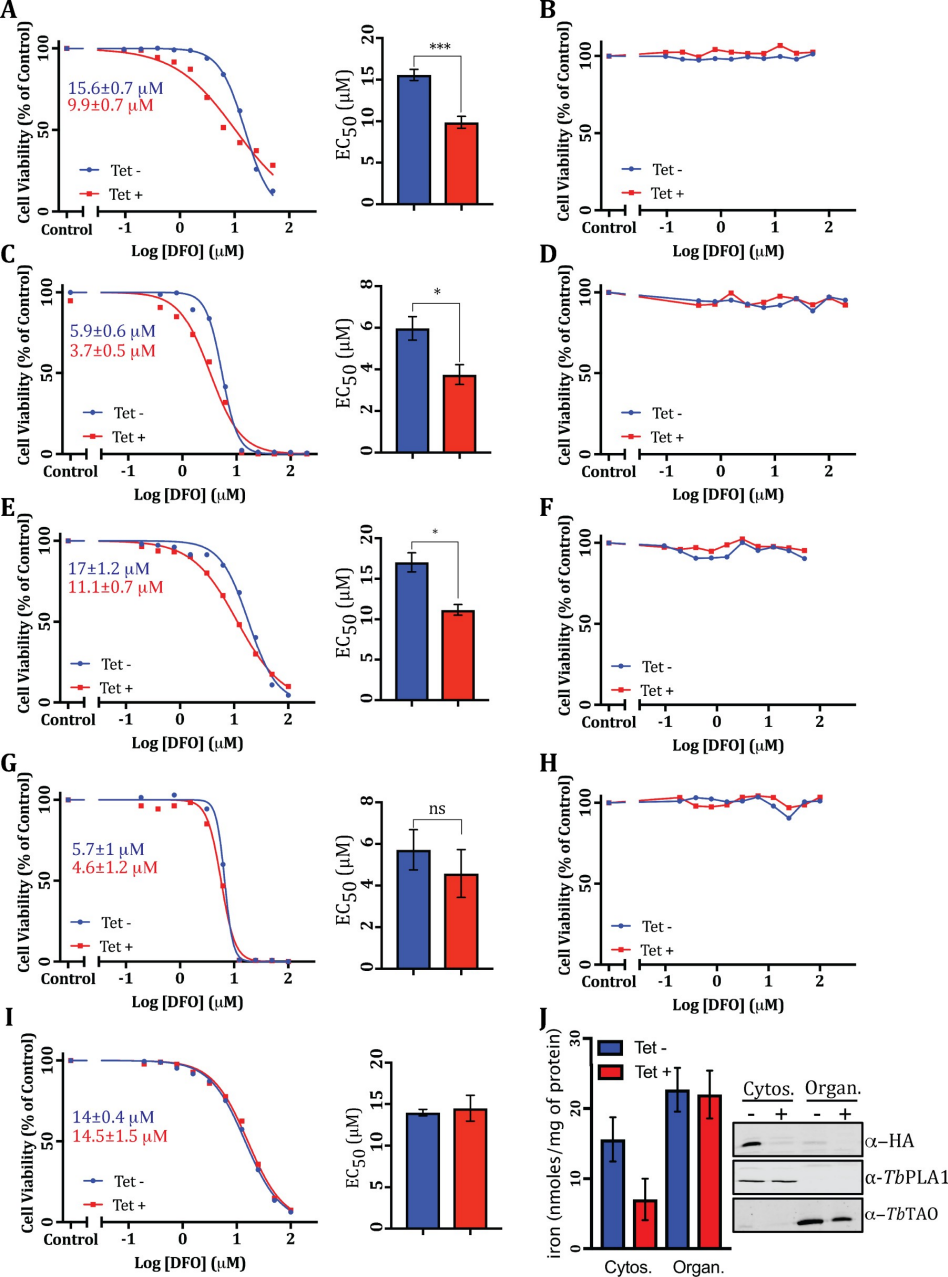
Knockdown of CIA members affects iron levels and sensitivity to iron depletion. Wild type (WT), *Tb*CIA2B, and *Tb*MMS19 RNAi cells were grown without (blue, Tet -) or with (red, Tet +) tetracycline for 24 hours and then treated with different concentrations of deferoxamine (DFO). After 2 or 3 days of incubation (PCF and BSF parasites, respectively), cell growth was measured by the Resazurin method for determination of EC_50_s. Representative DFO concentration-response curves are shown in (A) *Tb*CIA2B PCF, (C) *Tb*CIA2B BSF, (E) *Tb*MMS19 PCF, (G) *Tb*MMS19 BSF, or (I) WT PCF. Representative plots of DFO pre-incubated with an excess of iron before adding to (B) *Tb*CIA2B PCF, (D) *Tb*CIA2B BSF, (F) *Tb*MMS19 PCF, (H) *Tb*MMS19 BSF. The values shown in the inset of the curves are the mean DFO EC_50_s for induced or uninduced cultures. The bar charts on the right side of the curves are the mean ± SEM EC_50_s of 3 independent experiments performed in quadruplicate. ns = non-significant; * = p< 0.05; *** = p<0.001 (two tailed paired t test). (J) PCF*Tb*CIA2B RNAi cells were grown for 4 days in the presence (red) or absence (blue) of tetracycline and the content of iron bound to proteins was measured in the cytosolic and organellar fractions of digitonin permeabilised parasites. The purity of the cellular fractions was validated by Western blot using anti-HA (*Tb*CIA2B-HA), anti-*Tb*PLA1 (cytosolic marker), or anti-*Tb*TAO (mitochondrial marker).

Since *Tb*MMS19 and *Tb*CIA2B exhibited essentiality in *T*. *brucei* (**Fig 2A** and **2C**), we addressed the influence of these CTC components on the iron metabolism of the parasite. For this purpose, we used deferoxamine (DFO), a siderophore that chelates Fe^3+^ but has no effect on iron bound to either haem or transferrin [38] and which starves the cells by sequestering the labile iron pool [39–41]. *Tb*CIA2B RNAi cell lines were induced with tetracycline (Tet) for 24 hours and then challenged with different concentrations of DFO for 2 or 3 days (PCF and BSF, respectively), when cell proliferation was measured. When depleted of *Tb*CIA2B, both life stages were significantly more susceptible to DFO compared to those with normal levels of this protein (**Fig 3A** and **3C**), suggesting a decrease in the pool of available intracellular iron. A similar effect was observed upon *TbMMS19* knock down in PCF cells (**Fig 3E**). Additionally, WT PCF cells grown in the presence of Tet and treated with DFO under the same conditions displayed identical EC_50_ values as those grown in the absence of the antibiotics (**Fig 3I**), confirming that this result was specifically due to *TbCIA2B* or *TbMMS19* knockdown and not to the synergistic effects of DFO and Tet, which is also a chelator of polyvalent metal cations [42]. In agreement with this finding, no DFO toxicity was observed when *Tb*CIA2B or *Tb*MMS19 RNAi parasites were treated with drug pre-saturated with an excess of Fe^3+^ (**Fig 3B, 3D, 3F and 3H**), strongly indicating that the enhanced sensitivity can be specifically attributed to iron depletion and not to off-target effects of DFO. Furthermore, ferene assays suggested that the content of iron bound to proteins in the cytosolic lysates of *TbCIA2B* knockdowns was lower than that found in uninduced cells, whereas protein-bound iron levels did not change in the organellar fractions (**Fig 3J**).

### Yeast complementation assay

**Fig 4:**
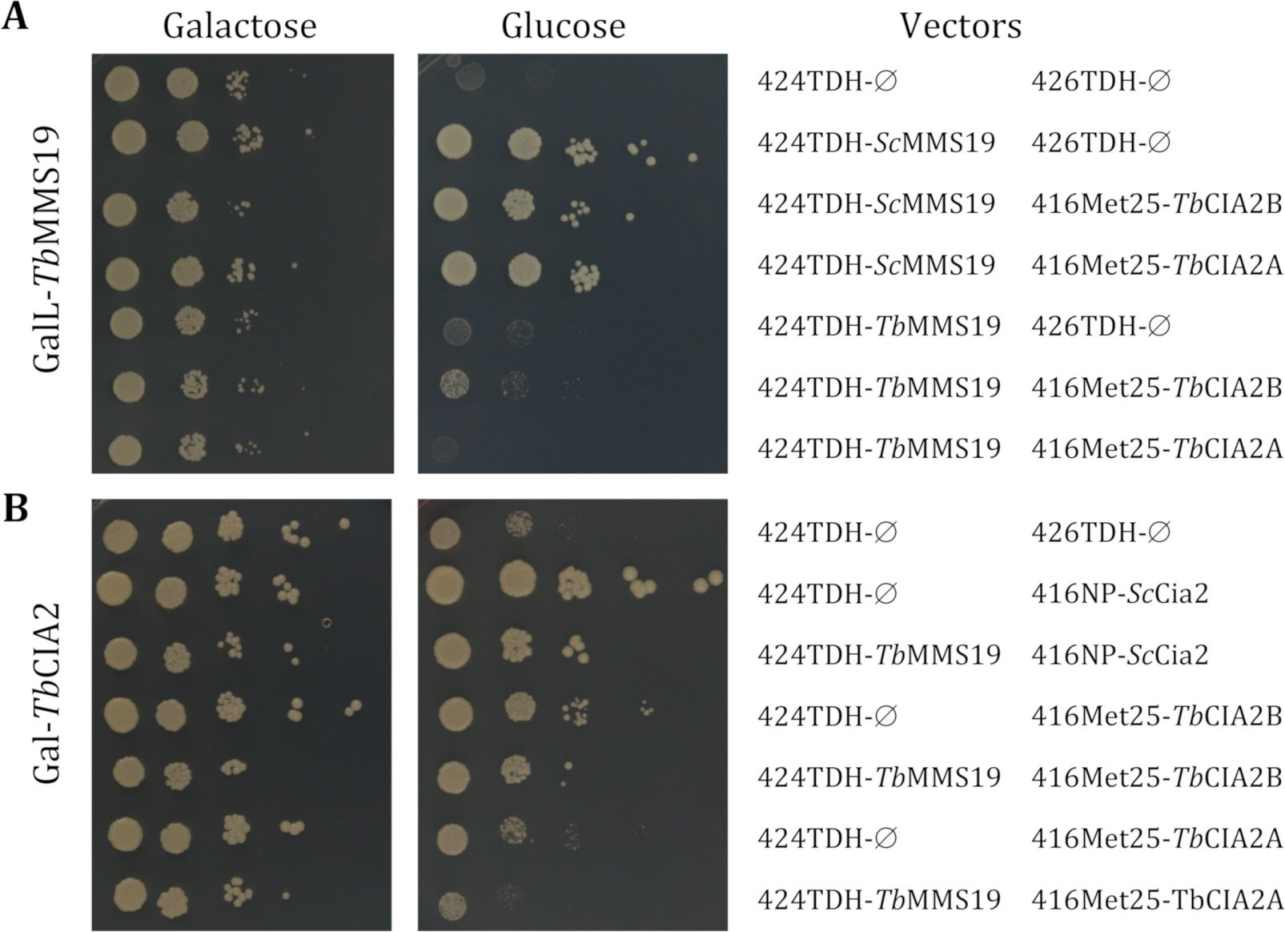
*Tb*CIA2B, but not *Tb*CIA2A, functionally replace yeast homologue *Sc*Cia2. Plasmids p424, p426 or p416, empty (Ø), or with the indicated genes, under the control of the strong promoters *MET25* or *TDH3*, and the natural promoter (NP) of *S*. *cerevisiae CIA2* were transformed into W303 cells, strains GalL-*MMS19* (A) and Gal-CIA2 (B). Cells were grown for 16 h in liquid minimal medium supplemented with glucose (2%). After washing, 10-fold serial dilutions were spotted onto agar plates containing minimal medium supplemented with galactose or glucose and incubated at 30°C for 2 days. The result was reproduced at least three times with independent transformations.

For functional complementation assays, *TbCIA2A*, *TbCIA2B* and *TbMMS19* were PCR-amplified from genomic DNA and cloned into yeast expression vectors under the control of the *TDH3* or *MET25* promoters of *Saccharomyces cerevisiae* [25,43]. Plasmids without insert or plasmids encoding endogenous yeast CIA genes were used as controls. Subsequently, these constructs were transformed into regulatable yeast Gal-CIA mutants, in which the expression of the cognate CIA gene is induced in the presence of galactose and repressed by the presence of glucose as described elsewhere [25]. The growth defect of Mms19-depleted cells on glucose-containing medium was not restored by *Tb*MMS19 expression, even when *Tb*MMS19 was co-expressed with either *Tb*CIA2A or *Tb*CIA2B (**Fig 4A**). Expression of *Tb*CIA2B partially rescued the growth of Cia2-depleted cells, but *Tb*CIA2A failed to do so (**Fig 4B**). For both *Tb*CIA2 proteins, co-expression with *Tb*MMS19 not only failed to enhance the rescue, but exhibited a dominant negative phenotype (**Fig 4**). Interestingly, when *Tb*MMS19 is co-expressed with Cia2 from *S*. *cerevisiae* (**Fig 4B**), the same dominant negative-like effect is observed. These findings show that *Tb*CIA2B can partially take over the role of its yeast counterpart, suggesting that it performs an orthologous function.

### Protein-protein interactions of the CTC

Individual interactions of the CTC proteins had only been mapped in detail for a few representatives of the eukaryotic supergroup Opisthokonta [44]. Moreover, the progress made in the field of Fe-S biology in the past decade suggests Fe-S proteins are diverse and abundant in a typical eukaryotic cell, but remained overlooked due to the difficulties related to their instability under aerobic conditions. To the best of our knowledge, the dynamics of protein-protein interactions of the CTC had not been studied in any Excavata, with only a few examples of identification and functional studies of CTC components [45]; despite ~0.6% of the annotated proteins of *T. brucei* being predicted to contain Fe-S clusters [46–48], its Fe-S proteome remains largely unexplored.

One of the most valuable tools that contributed to expanding the list of mammalian Fe-S proteins was the use of mass spectrometry (MS) and affinity purifications to detect potential Fe-S proteins interacting with the human CIA targeting complex [13,14,49]. Therefore, aiming to gain insight into the composition of the *T*. *brucei* CTC and its interactions with cytosolic and nuclear Fe-S proteins, three complementary strategies for affinity purification/MS were devised: (i) *in situ* PTP-tagged CTC members in PCF were affinity purified by a two-step approach [50], (ii) V5-tagged CTC members in PCF and iii) V5-tagged CTC members in BSF were immunoaffinity purified using a technique suited for the detection of transient and/or weak interactions [51]. In all cases, MS detected proteins co-purifying with the tagged baits.

**Fig 5:**
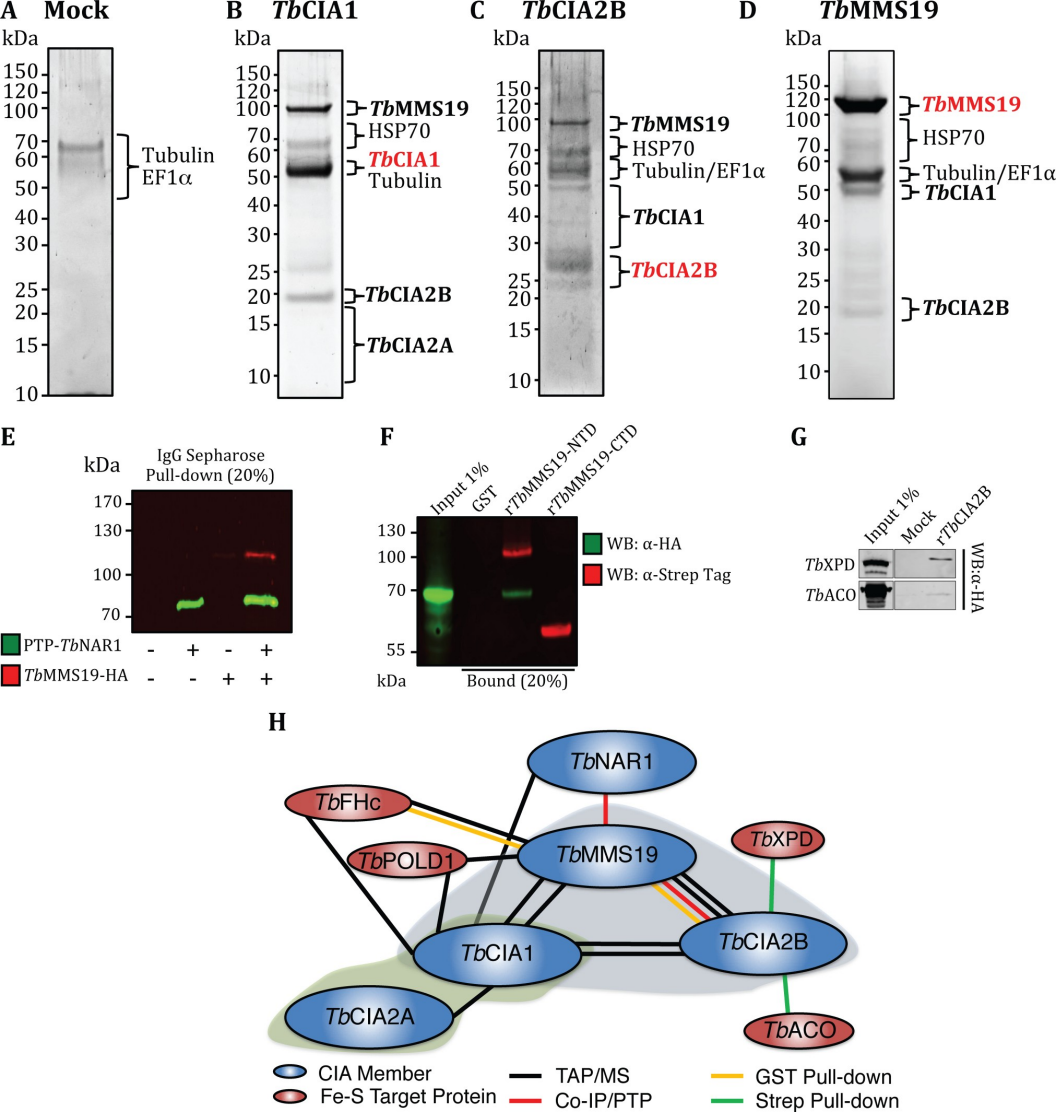
Protein-protein interaction profile of the CIA targeting complex. (A)-(D) Tandem affinity purification from WT parasites (mock) or PTP-tagged CIA components. Eluates were resolved in Bis-Tris gels and sections were analysed by mass spectrometry for protein identification. (E) Co-IP of PTP-*Tb*NAR1 with *Tb*MMS19-HA. Lysates of double tagged PCF parasites were incubated with IgG-Sepharose and the bound material subjected to SDS-PAGE and immunostaining with anti-HA and anti-protein A (PTP) antibodies. (F) Pull-down of *Tb*FHc-HA from PCF *T*. *brucei* extracts by GST alone, r*Tb*MMS19-NTD, r*Tb*MMS19-CTD. (G) Pull-down of *Tb*XPD-HA, or *Tb*ACO-HA from *T*. *brucei* PCF extracts by HIS-tagged r*Tb*CIA2B. The bound material of the pull-downs was resolved by SDS-PAGE and immunostained with anti-HA, anti-Strep Tag II, or anti-HIS antibodies. (H) Schematic representation of the protein-protein interaction profile of the *T*. *brucei* CIA targeting complex. The shaded blue bubble represents the identified members of the canonical CIA targeting complex and the green bubble represents the binary complex formed by *Tb*CIA2A-*Tb*CIA1.

Tandem affinity purifications were performed using PTP-*Tb*CIA1, PTP-*Tb*CIA2B, or *Tb*MMS19-PTP as baits, and mock purifications with wild type PCF parasites served as negative controls. Unfortunately, we were not able to purify PTP-*Tb*CIA2A complexes by this method for reasons that remain unclear, but may be related to the PTP tag (~19 kDa) being larger than *Tb*CIA2A, which is a protein of ~17 kDa. SYPRO Ruby stained SDS-PAGE gels of the final PTP elutions are shown in **Fig 5A–5D**. This exercise revealed that PTP-tagged CTC components can be reciprocally co-purified, proving the existence of the canonical ternary complex (*Tb*CIA1-*Tb*MMS19-*Tb*CIA2B). In *Tb*CIA2A-V5 pull-down assays (**S3 and S4 Tables**), *Tb*CIA1, but not *Tb*CIA2B or *Tb*MMS19, was found interacting with the bait protein, in a configuration reminiscent of that described for the human CTC [13–15]. Abundant proteins such as tubulins and the eukaryotic elongation factor 1α (EF1α) were present in control PTP purifications, but no detectable levels of CIA proteins were seen in these samples (**Fig 5A**). A summary of the PTP/MS data for the co-purified CTC members is shown in **S2 Table**. In addition to this, affinity pull-downs with V5-tagged *Tb*CIA1, *Tb*CIA2A, *Tb*CIA2B, and *Tb*MMS19 in PCF and BSF confirmed the reciprocal nature of the interactions and the presence of the similar complex configurations in both life stages (**S3 and S4 Tables**), and also showed in PCF that *Tb*CIA1, *Tb*CIA2A and *Tb*MMS19, but not *Tb*CIA2B, co-immunoprecipitated with *Tb*NAR1, the upstream CIA component that mediates the transfer of Fe-S clusters from the early-acting part of the pathway to the CTC (**S3 Table)**. Moreover, co-IP performed with lysates of a double-tagged strain of PCF parasites co-expressing PTP-*Tb*NAR1 and *Tb*MMS19-HA further validated this interaction (**Fig 5E**).

Next, in an attempt to identify potential target Fe-S proteins, we investigated other proteins co-eluting with members of the CTC. The combined TAP/MS and co-IP experiments identified over 200 such proteins, most of them in association with *Tb*MMS19 and/or *Tb*CIA1 (**S5 Table**). To inquire if these proteins were known or could be predicted to contain Fe-S clusters, the amino acid sequences of the hits were retrieved from the TriTryp database [46] and analysed with MetalPredator [48], a tool to predict Fe-S clusters in polypeptide chains based on the presence of known Fe-S domains and metal-binding motifs. This analysis returned three positive hits: the catalytic subunit of Pol δ (*Tb*POLD1, Tb927.2.1800), the class I cytosolic fumarate hydratase (*Tb*FHc, Tb927.3.4500), and a putative radical SAM tRNA modification enzyme (Tb927.6.3510) (**S5 Table)**.

In order to further examine the interactions of the CTC with Fe-S proteins, the amino- or carboxy-terminal domains of *Tb*MMS19 (respectively, recombinant (r) *Tb*MMS19-NTD and r*Tb*MMS19-CTD) were expressed in *E*. *coli* as GST-Strep-Tag II fusion proteins. Equimolar amounts of purified recombinant proteins or glutathione S-transferase (GST), used as a negative control, were coupled to glutathione Sepharose 4B beads, incubated with soluble extracts of PCF parasites expressing HA-tagged *Tb*FHc, and the interactions were assessed by Western blotting. This pull-down confirmed the interaction detected by TAP/MS and further showed that *Tb*FHc was able to interact with r*Tb*MMS19-NTD, but not r*Tb*MMS19-CTD or GST alone (**Fig 5F**). Also, a relatively low number of proteins were detected in PCF TAP/MS or V5 co-IP/MS experiments with *Tb*CIA2B (**S5 Table**). In order to verify possible protein-protein interactions of *Tb*CIA2B that could not be detected by other methods, this protein was expressed in *E*. *coli* as a fusion with an N-terminal Strep-Tag II and a C-terminal hexahistidine tag (r*Tb*CIA2B), then immobilised to Strep-Tactin Sepharose and incubated with extracts of parasites expressing either tagged aconitase (*Tb*ACO-HA) or the DNA helicase XPD (Xeroderma pigmentosum group D homologue,*Tb*XPD-HA). As shown in **Fig 5G**, *Tb*XPD interacts with r*Tb*CIA2B. Moreover, *Tb*ACO also interacted with r*Tb*CIA2B (**Fig 5G**). This is in accordance with the results depicted in **Fig 2G** and **2I**, where the silencing of *Tb*CIA2B led to decreased cytosolic activity of *Tb*ACO. These results validate the position of the CTC as the late-acting module of the CIA machinery at the interface between the upstream *Tb*NAR1 and the client Fe-S proteins.

A summary of interactions detected by TAP/MS experiments and additionally confirmed by co-IPs is depicted in **Fig 5H**. The interaction with *Tb*NAR1 was not observed in the V5-tagged co-immunoprecipitations performed in BSF trypanosomes (**S4 Table**). This difference may reflect stage-specific requirement of the CIA pathway. Importantly, mutual interactions of the CTC members are the same in the BSF and PCF cells, although the sets of their targets differ from one another and require further analysis to determine their capability to bear an Fe-S cluster.

Interestingly, several proteins captured by the PCF TAP/MS methodology show multiple clustered cysteine residues, including Cys-Pro dipeptide sequences (Tb927.3.4360, Tb927.7.4390, Tb927.2.5130 and Tb927.8.4890). We are currently pursuing the possibility that these proteins harbour an Fe-S cluster by heterologous expression and purification.

### The CTC is assembled at the C-terminal domain of *Tb*MMS19

Aiming to better understand the dynamics of the interactions amongst the CTC members, it was of interest to identify the site through which members of this complex interact. However, the amino acid sequence of *Tb*MMS19 is poorly conserved when compared to its human or yeast homologues [52], and no crystal structures of this protein have been elucidated so far. Nevertheless, *in silico* homology modelling of the tertiary structure of *Tb*MMS19 using the Phyre2 server [53,54] suggested the overall architecture of an Armadillo-like protein that contains α-helical HEAT repeat motifs throughout its sequence [55,56], as previously predicted for MMS19 in higher eukaryotes [57]. In human cells, CIA2B and CIAO1 interact with the tightly spaced HEAT repeats at a region of the MMS19 C-terminal domain [58], whereas in *Tb*MMS19 these motifs seem to be more loosely distributed and are more numerous (**S3 Fig**). We hypothesised that the binding site for the CIA proteins would be different in trypanosomes, given the divergent amino acid composition and apparent different distribution of the repeats.

**Fig 6:**
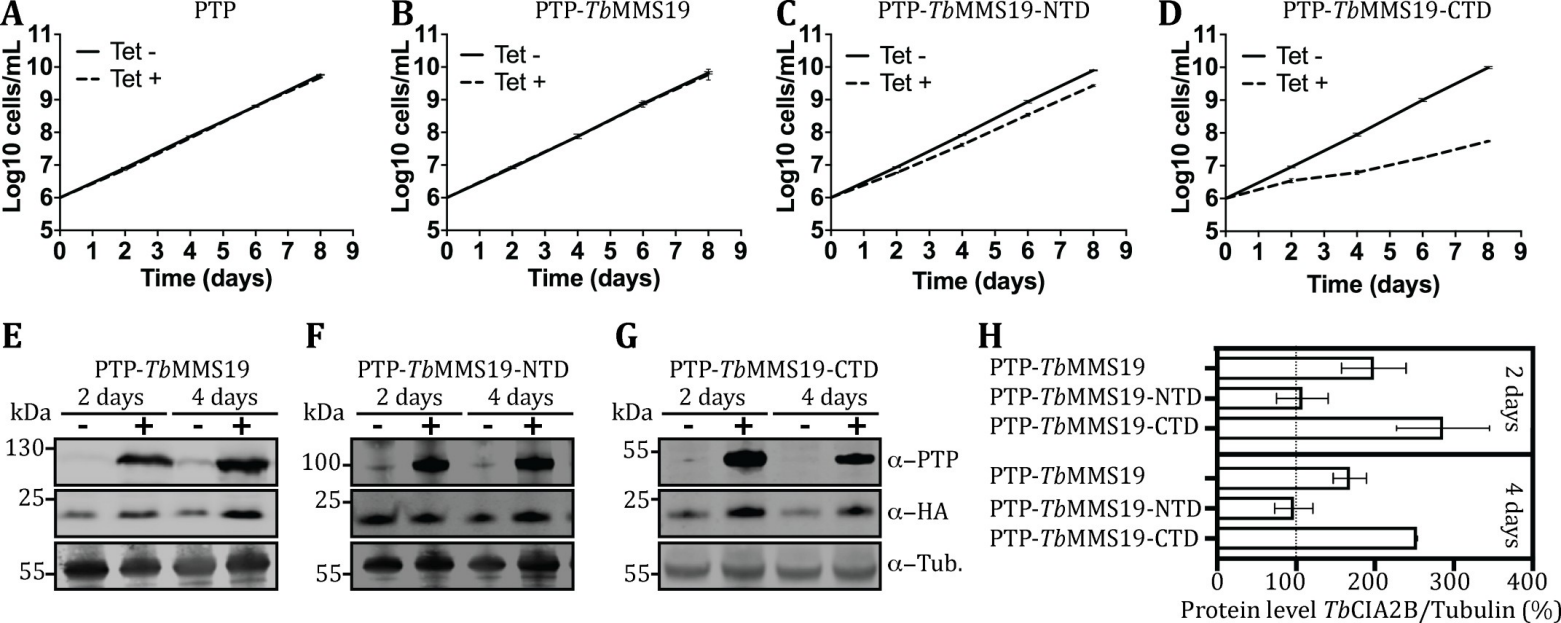
Overexpression of the C-terminal domain of *Tb*MMS19 is detrimental for cell growth and increases *Tb*CIA2B levels. Growth curves of PCF *T*. *brucei* carrying an *in situ* HA-tagged copy of *Tb*CIA2B and overexpressing an ectopic inducible copy of (A) PTP tag, (B) PTP-MMS19, (C) PTP-MMS19-NTD, or (D) PTP-MMS19-CTD. Cell numbers were assessed in the presence (Tet +) or absence (Tet -) of tetracycline in the culture medium for the specified number of days. Data points represent the mean ± SD of 2 independent experiments. (E)-(G) Parasites were grown for 2 or 4 days in the presence or absence of tetracycline. Total cell lysates were probed by Western blot using anti-Protein A (PTP-tag), anti-HA and anti-tubulin antibodies. (H) Protein expression was calculated by densitometry and the HA/tubulin ratio in induced cells was normalised to the respective ratio of uninduced cultures (dashed line). Bars represent the mean ± SEM of two experiments.

To clarify this question, *Tb*CIA2B-HA cell lines of PCF parasites were transfected with constructs derived from the pLEW82 vector that integrates into the non-transcribed spacer of the *rDNA* locus and allows strong ectopic overexpression of proteins in the presence of Tet [21]. We engineered cell lines in which the PTP-tagged full-length *Tb*MMS19 (PTP-*Tb*MMS19), its N-terminal (PTP-*Tb*MMS19-NTD) or C-terminal domain (PTP-*Tb*MMS19-CTD) can be conditionally overexpressed. Both the overexpression of the control PTP tag alone and PTP-*Tb*MMS19 did not impact the cell growth (**Fig 6A** and **6B**, respectively). However, overexpression of PTP-*Tb*MMS19-NTD caused a mild cell growth delay (**Fig 6C**), whereas a prominent dominant-negative phenotype developed when excess PTP-*Tb*MMS19-CTD was produced, causing near-arrest of the cell growth after two days in culture (**Fig 6D**). Interestingly, overexpressing PTP-*Tb*MMS19 or PTP-*Tb*MMS19-CTD for two days resulted in ~2 and ~3-fold higher levels of *Tb*CIA2B-HA, respectively, and this effect was sustained after 4 days of overexpression (**Fig 6E, 6G** and **6H**). Conversely, in cells induced to overexpress PTP-*Tb*MMS19-NTD, the levels of *Tb*CIA2B-HA remained unaltered (**Fig 6F** and **6H**).

**Fig 7:**
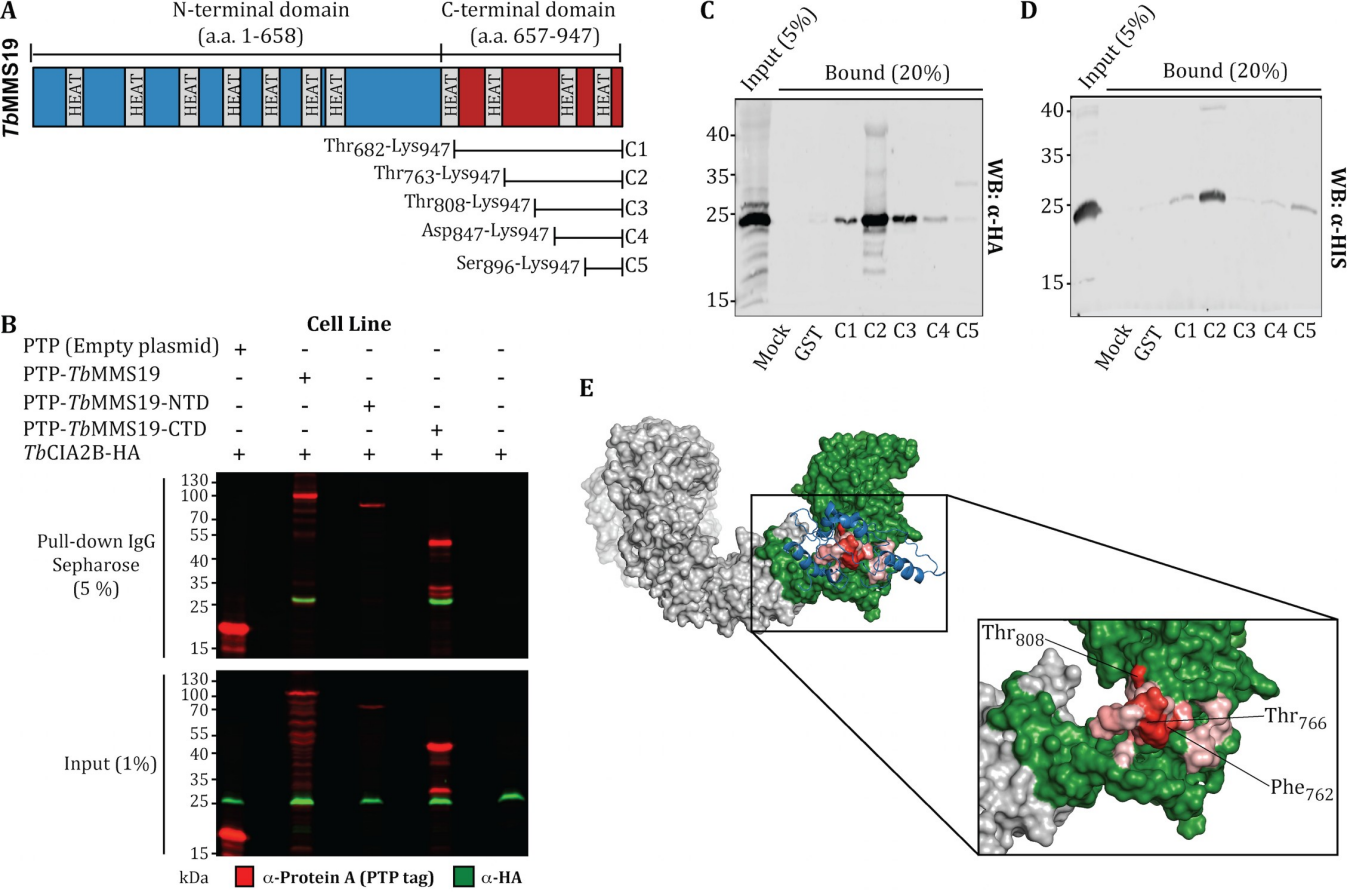
The C-terminal domain of *Tb*MMS19 is the binding site of *Tb*CIA2B. (A) Schematic representation of *T*. *brucei* MMS19. The N-terminal domain is depicted in blue and the C-terminal domain in red. Grey boxes show the position of the 11 HEAT repeats identified in *Tb*MMS19. C1-C5 represent the position of GST/Strep-tagged recombinant fragments of *Tb*MMS19-CTD used in pull-down experiments. (B) Co-IP of *Tb*CIA2B with *Tb*MMS19. Lysates of *Tb*CIA2B-HA cells overexpressing the PTP tag (empty vector), PTP-*Tb*MMS19, PTP*-Tb*MMS19-NTD, or PTP-*Tb*MMS19-CTD were incubated with IgG Sepharose. The bound proteins were subjected to Western blot and probed with Anti-HA and Anti-Protein A (PTP) antibodies. Identification of the *Tb*CIA2B binding site at *Tb*MMS19. Cell extracts of *T*. *brucei* expressing HA-tagged *Tb*CIA2B (C), or r*Tb*CIA2B (D) were incubated with GST/Strep-tagged fragments (C1-C5) immobilised in glutathione Sepharose beads. Bound material was resolved by SDS-PAGE and immunoblotted with anti-HA or anti-HIS tag antibodies as indicated. (E) The 3D structures of *Tb*MMS19 and *Tb*CIA2B were modelled by homology using the Phyre2 server. The grey and green surfaces represent respectively *Tb*MMS19-NTD, and *Tb*MMS19-CTD while the blue ribbon corresponds to *Tb*CIA2B. The *Tb*MMS19-*Tb*CIA2B complex was modelled *in silico* with ClusPro and correctly predicted that *Tb*CIA2B binds to *Tb*MMS19-CTD. This theoretical complex was analysed with PredHS to identify *Tb*MMS19 residues at the surface of interaction with *Tb*CIA2B. The surface of interaction is represented in a red scale according to their PredHS SVM-Hot spot score. Labelled residues at the inset had the highest SVM-Hot spot scores. PyMol (Schrödinger, LLC) was used to visualise and generate the figures. The binding site of TbCIA2B was found to be in a region comprising ~185 amino acids at the C-terminal domain of *Tb*MMS19.

To investigate if the up-regulation of *Tb*CIA2B by *Tb*MMS19 or its C-terminal domain was dependent on their interaction, we performed co-IPs with extracts of parasites induced overnight to overexpress PTP-tagged *Tb*MMS19, *Tb*MMS19-NTD or *Tb*MMS19-CTD. Cells overexpressing only the PTP-tag or those not transfected with pLEW82 constructs were used as controls. Although lower levels of PTP-*Tb*MMS19-NTD were observed when compared to those achieved for PTP-*Tb*MMS19 or PTP-*Tb*MMS19-CTD after induction, co-IP assays indicated that *Tb*CIA2B-HA was able to bind PTP-*Tb*MMS19 and PTP-*Tb*MMS19-CTD, but not PTP-*Tb*MMS19–NTD (**Fig 7B**). Additional TAP/MS assays revealed that *Tb*CIA1 was only detected in eluates from PTP-*Tb*MMS19 and PTP-*Tb*MMS19-CTD, but not PTP-*Tb*MMS19-NTD (**S4 Fig**), which implicates that *Tb*MMS19-CTD acts as a docking site for the assembly of the ternary complex.

To pinpoint the binding site of *Tb*CIA2B within *Tb*MMS19-CTD, we used recombinant fragments of the latter (named C1-C5, **Fig 7A**), expressed as GST-Strep-Tag II fusions. Equimolar amounts of C1-C5 or GST were bound to glutathione Sepharose 4B beads and incubated with cellular extracts of *T*. *brucei* expressing HA-tagged *Tb*CIA2B (**Fig 7C**) or purified r*Tb*CIA2B (**Fig 7D**). Fragments C1-C4 were able to bind *Tb*CIA2B-HA from cell lysates, although fragment C2 appears to bind with higher affinity, while the C5 fragment, containing only 1 HEAT domain, has very weak affinity (**Fig 7C**). Moreover, fragments C1, C2, C4 and C5 captured purified r*Tb*CIA2B and also in this case, C2 displayed the highest binding capacity (**Fig 7D**). Curiously, C1 was less capable of binding to *Tb*CIA2B than the smaller C2 or C3 fragments. One possible explanation is that C1 adopted a fold that hinders the ability of *Tb*CIA2B to reach the interaction surface which could, in turn, be more accessible in C2. We also observed that the C3 and C4 fragments have an enhanced ability to bind *Tb*CIA2B in cell extracts in comparison to the purified recombinant protein, hinting at the presence of a factor that stabilises the complex. The C5 fragment interacted (albeit weakly) with recombinant and native *Tb*CIA2B (**Fig 7C and 7D**), suggesting that this repeat is the minimal structural unit necessary to form the *Tb*MMS19-*Tb*CIA2B complex. This fragment contains 50 amino acids that roughly correspond to the most C-terminal HEAT repeat in *Tb*MMS19, although such interaction likely spans a much larger contact surface, involving at least two HEAT repeats localized between the residues Val_763_ and Lys_947_ of *Tb*MMS19.

Given the paucity of structural analyses for the CIA proteins, individually or in a complex, the 3D structures of *Tb*MMS19 and *Tb*CIA2B were modelled by homology using the Phyre2 server [53]. The predicted structures were subsequently used to generate models of protein-protein interaction by *in silico* docking with ClusPro [59]. Corroborating our experimental findings, the top scoring model for the *Tb*MMS19-*Tb*CIA2B complex correctly predicted that *Tb*CIA2B should bind to *Tb*MMS19–CTD (**Fig 7E**). In fact, most of the top scoring models also pointed to the binding site of the C-terminal domain of *Tb*MMS19 (**S5 Fig)**. Given this reassuring overlap between the experimental data and *in silico* predictions, we aimed to refine this analysis by examining the best complex model with PredHS, a tool that integrates analysis of structural and energetic properties to identify regions at the contact surface, which are more likely to be crucial for protein-protein interactions (i.e. hot spots or hot regions of interaction) [60,61]. This analysis suggested that although Thr_808_ seems to be important, a contiguous region of 12 amino acids in the *Tb*MMS19-CTD (Phe_762_-Thr_773_) could be essential for the interaction with *Tb*CIA2B. These residues are depicted in a scale of red in **Fig 7E**. This model fits satisfactorily our experimental data, which indicated that the C2 fragment (Val_763_-Lys_947_) binds tightly to r*Tb*CIA2B, while the C3 fragment (Thr_808_-Lys_947_) interacted (very) weakly with it, although this association was stabilised when the native protein was present in cell lysates. Collectively, these results indicate that *Tb*CIA2B binds directly and tightly to the C-terminal domain of *Tb*MMS19, but this interaction is likely to require additional factors to stabilise the complex.

## Discussion

Since the subcellular localisation of the CIA components seems to depend upon the organism under study [34,62–66], we aimed to clarify the cellular compartment in which the late-acting module of the CIA machinery was present in trypanosomes. For this aim, a combination of immunofluorescence and immunoblot analyses of detergent-permeabilised cell extracts localised all four studied proteins to the cytosol, in agreement with data from mammalian cells [13,15,67]. However, Mms19 in *Schizosaccharomyces pombe*, and Cia1 in *S*. *cerevisiae* are predominantly nuclear [34,68]. In the plant *Arabidopsis thaliana*, MMS19 is exclusively cytosolic, although other members of the CTC exist both in the nuclear and cytosolic compartments [66]. Moreover, *Giardia intestinalis* exhibits a dual localisation of Cia2, between the intermembrane space of the mitosome and the cytosol [45].

*Tb*MMS19 and *Tb*CIA2B were shown to be essential for the survival of PCF, but their depletion exhibited only marginal defects in BSF trypanosomes. In human cells, the levels of CIA2B are greatly reduced when MMS19 is ablated [10,12,49], yet MMS19 remains steady regardless of the absence of CIA2B, suggesting a tight regulation of CIA2B rather than reciprocal stabilisation between the interacting partners, since MMS19 prevents proteasomal degradation of CIA2B in a binding-dependent manner [49]. Interestingly, overexpressing the C-terminal domain of *Tb*MMS19 produced a dominant-negative phenotype with severe defects on the cell growth and concomitant up-regulation of the *Tb*CIA2B levels. One plausible explanation for this finding concerns the modes of interaction within the CTC, since the C-terminal domain of *Tb*MMS19 appears to be the docking site of the targeting complex, as recently described also for human cells [58]. It is possible that high levels of this truncated protein can sequester *Tb*CIA2B, *Tb*CIA1, as well as client proteins into non-functional complexes, thus depleting the cell of at least two CTC members and mimicking the effect of a double knockdown. On the other hand, the depletion of *Tb*CIA2A does not affect PCF, and only has a mild effect in BSF. Though the MS data suggests non-redundant functions, such as the formation of different subcomplexes among various components of the CTC, the growth phenotype in the RNAi cell lines, as well as the capability of infection of BSF RNAi cell lines, hint at the possibility of function overlapping. However, residual proteins escaping RNAi knockdown may be sufficient to maintain the functionality of the CIA machinery.

The effect of RNAi-mediated depletion of the late-acting CIA factors was monitored through the activity of *Tb*ACO. The CIA2A protein aids the maturation of iron regulatory protein 1 (IRP1), the human homologue of *Tb*ACO, and stabilizes IRP2 by Fe-S independent mechanisms, whereas CIA2B has a role in the maturation of numerous cytosolic and nuclear Fe-S proteins [15]. Conversely, the CIA proteins do not exert a direct impact on iron regulation in *S*. *cerevisiae*, and an IRP1-like mechanism has not been implicated in *T*. *brucei* iron regulation [32,69] Regardless, *Tb*MMS19 and *Tb*CIA2B were found to be essential for the activity of the cytosolic but not the mitochondrial fraction of this enzyme. This is in line with previous studies, which demonstrated that the mitochondrial pool of this enzyme is matured by the ISC pathway, the mitochondrial machinery for Fe-S biogenesis [70–72], while the cytosolic fraction requires both the ISC and CIA machineries to obtain its cluster [25,73]. Furthermore, *Tb*ACO was shown to interact with *Tb*CIA2B in dedicated pull-down assays. The growth complementation of Cia2-depleted yeast cells by *Tb*CIA2B presents independent evidence for the functional conservation of this protein in the CTC. Taken together, the functional and physical interactions of the CTC with *Tb*ACO provide an example of a maturation mechanism of cytosolic Fe-S proteins in *T*. *brucei*.

We observed that upon silencing of *Tb*CIA2B or *Tb*MMS19, PCF cells displayed an enhanced sensitivity to the iron chelator deferoxamine, with EC_50_ values about 1.5 times lower than in uninduced controls. The specificity of this effect was confirmed by incubating trypanosomes with deferoxamine pre-saturated with iron, which abolished its toxicity. Furthermore, BSF parasites depleted of *Tb*CIA2B also displayed equally lower EC_50_ values. This effect was consistent, although not as pronounced as in conditional null mutants of the cation channel mucolipin 1 that delivers iron to the cytosol of BSF flagellates [74]. Although IRP1-like mechanisms implicating the CIA machinery in iron sensing and regulation, such as those described in human cells [15], seem unlikely to exist in *T*. *brucei* [32,69], a role for unknown Fe-S cluster-containing factors in iron regulation cannot be completely ruled out. Deferoxamine acts by scavenging the cellular labile iron pool (LIP), thus preventing incorporation of this element into the newly synthesised apo-proteins [75]. The precise composition of LIP is uncertain, but free iron is seldom present in the intracellular milieu, given its capacity to generate reactive oxygen species via the Fenton reaction [26,76]. The source of iron for the assembly of Fe-S clusters in the cytosol remains unknown, although one line of thought speculates that the scaffold proteins for Fe-S cluster assembly can bind LIP directly [77]. If this was the case, LIP depletion by deferoxamine would magnify an already impaired CIA function in cells depleted of *Tb*CIA2B or *Tb*MMS19, thus explaining the increased sensitivity. The LIP is expected to account for 0.2 to 3% of total cellular iron, with its bulk bound to the cytosolic and/or mitochondrial proteins [78]. Lower levels of protein-bound iron were observed in the cytosol of PCF flagellates depleted of *Tb*CIA2B but remained unchanged in organellar fractions, indicating that Fe-S proteins may comprise a considerable portion of the cytosolic iron content in *T*. *brucei*.

Collectively, these data demonstrate that the CTC is essential for the survival of *T*. *brucei in vitro* but does not seem to have an influence on the pathogenicity of the parasite in *in vivo* mouse experiments. The CTC further functions in both the iron metabolism and the maturation of target Fe-S proteins. However, the processes of DNA damage repair appear to be more resilient to the depletion of the CTC in this excavate protist when compared to other eukaryotic systems, where they are strongly linked to the functionality of Fe-S assembly pathways [13,14,35–37]. This observation can be partially attributed to the unique mechanisms of nucleotide excision repair (NER) utilised by this parasite. In yeast and humans, XPD is part of the transcription factor complex TFIIH [79]. Along with XPB, XPD forms the core of this complex, acting together in transcription initiation and DNA repair [79]. In *T*. *brucei*, *Tb*XPD and *Tb*XPB are not part of the same complex [80], nor do they respond to DNA damage in the same fashion [80,81]. Moreover, XPB exhibits two orthologues in this flagellate, known as *Tb*XPB and *Tb*XPB-R (or *Tb*XPBz), of which only the latter seems to be involved in NER independently of TFIIH [80,81]. Yet, contrary to yeast and humans, *Tb*XPD knock-downs in PCF and BSF exhibit different growth phenotypes, the protein does not influence NER proficiency and seems to be mostly involved in transcription initiation [80,81]. The genetic, functional and physical interactions of XPD (Rad3 in yeast) with the late-acting members of the CIA machinery have been well described in various organisms, and the ternary CTC is necessary for efficient maturation of this protein [12,35,36,82]. Interestingly, *Tb*XPD was undetectable in our TAP/MS and V5 co-IP/MS assays, which could suggest a lower affinity of this transient association with the CTC in *T*. *brucei* than that observed for its human and yeast counterparts, although an interaction with *Tb*CIA2B was seen in a dedicated pull-down assay. It is also plausible that down-regulation of the CIA machinery can trigger compensatory mechanisms of DNA repair, which are Fe-S independent. An alternative explanation is that residual levels of the CTC components upon RNAi knockdown may be sufficient to maintain adequate levels of maturation of Fe-S proteins involved in DNA repair.

We used a combination of TAP/MS, co-IP/MS and dedicated pull-downs to detect potential client Fe-S proteins of the CTC. This approach validated the interactions amongst the late-acting members of the CIA machinery. A relatively large number of proteins was found in (transient) association with the CTC, with only a few of them predicted to contain Fe-S clusters. These analyses revealed that the three core components of the canonical ternary CTC could indeed be reciprocally co-purified showing that the CTC is conserved to both life stages of *T*. *brucei*. Interestingly, in both PCF and BSF cells, *Tb*CIA2A was only observed in complexes purified from PTP-*Tb*CIA1 or *Tb*CIA1-V5, while *Tb*MMS19 and *Tb*CIA2B were not detected in co-IPs with V5-tagged *Tb*CIA2A (**S2, S3 and S4 Tables**). Consistent with our findings, CIA2A was not reported as a core CTC member in the seminal studies that established the role of the ternary complex CIAO1-MMS19-CIA2B in the maturation of Fe-S proteins [13,14]. However, in HeLa cells CIAO1 was shown to associate with both CIA2A and CIA2B in a mutually exclusive fashion, with these complexes interacting selectively with distinct subsets of target proteins [15]. The lack of interaction, between *Tb*CIA2A and either *Tb*CIA2B or *Tb*MMS19 indicates the existence of a binary complex comprised of *Tb*CIA1 and *Tb*CIA2A in a configuration that is reminiscent of that described in mammalian cells [15], although the biological purpose of *Tb*CIA2A or the complex it forms with *Tb*CIA1 remains elusive at this time. It is worth mentioning here that a very weak interaction between *Tb*CIA2A and *Tb*MMS19 was detected in the BSF cells.

**Fig 8:**
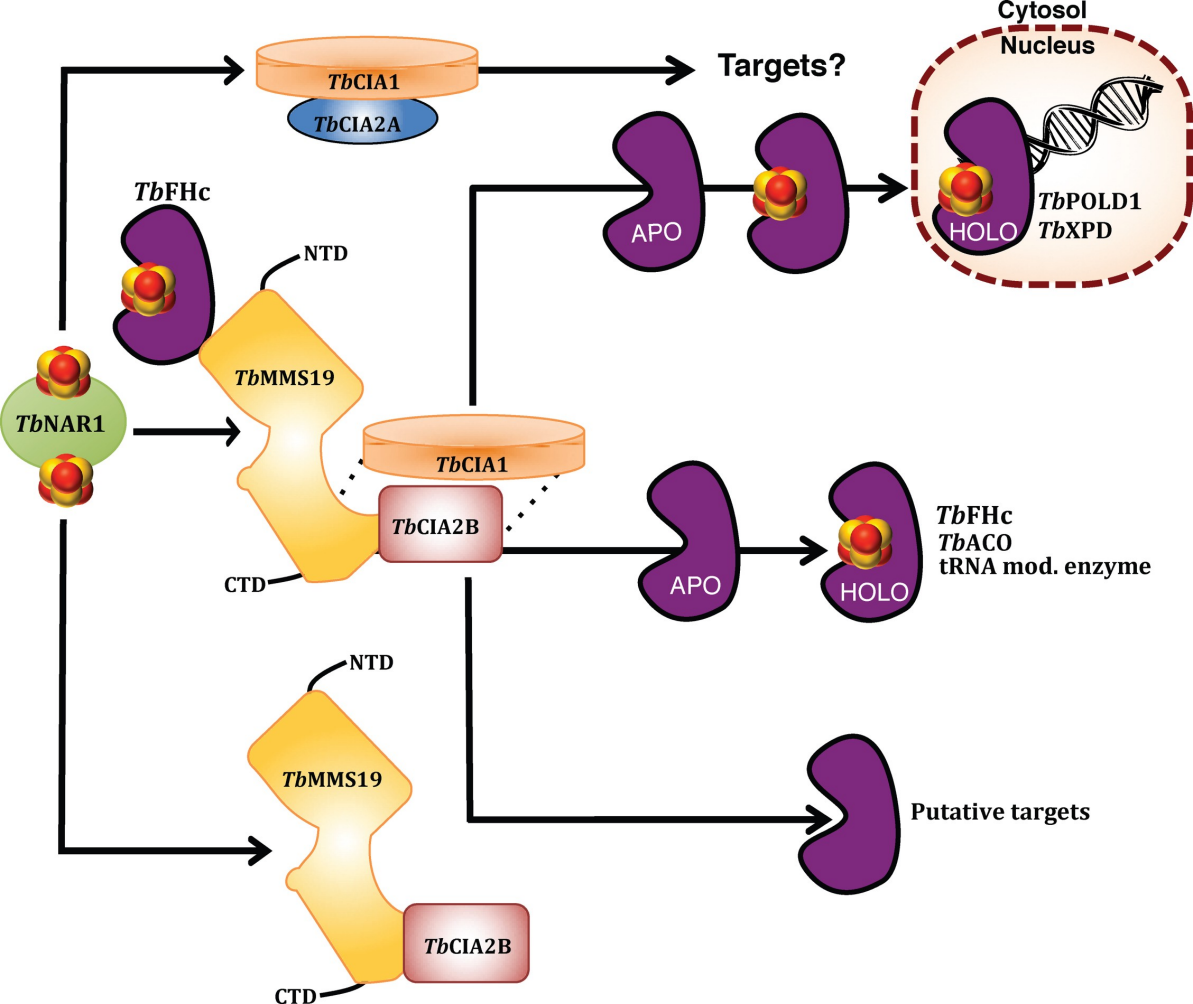
Functional model of the CIA targeting complex of *T*. *brucei*. *Tb*NAR1 receives a Fe-S cluster from the early-acting CIA machinery and interacts with members of the CIA targeting complex (CTC). *Tb*CIA2B binds tightly to the C-terminal domain of *Tb*MMS19 in and interaction possibly stabilised by *Tb*CIA1 (as indicated by the dotted lines), forming the canonical ternary CTC, although binary complexes also exist. The function of the complex formed by *Tb*CIA1 and *Tb*CIA2A is unknown. The targeting complex directly interacts with cytosolic and nuclear Fe-S proteins. *Tb*POLD1 was found in association with *Tb*CIA1 and *Tb*MMS19, and *Tb*XPD with *Tb*CIA2B. *Tb*FHc interacts with *Tb*MMS19 at its N-terminal domain and also co-purifies with *Tb*CIA1. *Tb*ACO interacts with *Tb*CIA2B but also requires *Tb*CIA1 for maturation. Other potential Fe-S proteins also interact with the complex, but their status as *bona fide* Fe-S proteins is unknown.

We demonstrate that *Tb*CIA2B interacts with the C-terminal domain of *Tb*MMS19. A schematic representation of the proposed model for the ternary *T*. *brucei* CTC is depicted in **Fig 8.** The C-terminal domain of *Tb*MMS19 (*Tb*MMS19-CTD) acts as a docking site for the other two members of the trimeric complex, namely *Tb*CIA2B and *Tb*CIA1. We can also conclude from our pull-down assays that *Tb*CIA2B independently interacts with *Tb*MMS19-CTD. In humans, the interaction between CIA2B and MMS19 has been shown to be vital not only for the stability of the CTC itself, but also for the association with client Fe-S proteins [58,83]. Interestingly, van Wietmarschen and colleagues [67] reported that *in vitro* translated murine CIA2B and MMS19 were not able to bind directly to each other, although both could interact with CIAO1. However, in support of our observations, Odermatt and Gari [58] showed that CIA2B binds to the C-terminal HEAT repeats of MMS19 in HeLa cells, and similar results were observed in pull-down assays with purified human proteins [83]. The status of *Tb*CIA1 in the CTC of *T*. *brucei* is less clear, since from our results we cannot distinguish whether its interaction with *Tb*MMS19 depends on the presence of *Tb*CIA2B. However, recombinant fragments of the C-terminal domain of *Tb*MMS19 had an enhanced ability to bind *Tb*CIA2B in cell extracts if compared to the purified recombinant protein. In agreement with these observations, human CIAO1 was reported to stabilise the interaction between CIA2B and the HEAT repeats at the C-terminal domain of MMS19, forming a trimeric complex [58]. Thus, we favour the interpretation that *Tb*CIA2B independently interacts with the C-terminal domain of *Tb*MMS19, yet this interaction may be further strengthened by other proteins, with *Tb*CIA1 being a prime candidate. Considering that both *Tb*CIA1 and *Tb*CIA2B are involved in the maturation of Fe-S proteins, it is possible that the assembly of the clusters into apo-proteins takes place at this C-terminal docking site [58].

The binding site of *Tb*CIA2B, as supported by *in silico* modelling of the*Tb*CIA2B-*Tb*MMS19 complex, was narrowed down to a region between the residues Val_763_-Lys_947_ of the C-terminal domain of *Tb*MMS19. The remarkable complementarity of the experimental observations with the *in silico* predictions allowed us to model the interface between the two proteins and identify residues likely involved in their interaction. However, bearing in mind that our data also strongly suggested the CTC exists in both binary and ternary versions, these simulations may not exactly reflect the whole scenario taking place at a cellular level. Since a reliable structural model for *Tb*CIA1 could not be generated, we did not attempt to dock a ternary *Tb*CIA2B-*Tb*CIA1-*Tb*MMS19 complex, or predict binary interactions of that protein. It is also important to recognise the caveats associated with this method, as homology-based structural models may not accurately reflect the minutia of biologically relevant conformations of proteins or complexes. Nevertheless, a similar approach has been successfully used to study the specificity of binding of the *trans*-acting acyltransferase to acyl-carrier proteins [84] and to design inhibitors of the human tumour necrosis factor [85]. Altogether, we believe our model provides a valuable snapshot of the *Tb*MMS19-*Tb*CIA2B interaction. The comprehensive analysis of protein-protein interactions for the CTC presented herein sheds light on the flexibility, as well as on the level of conservation of this ubiquitous eukaryotic pathway.

## Materials and methods

### Parasite cultivation and transfection

*T*. *brucei* PCF 29–13 [21], and SmOxP927 [86] cell lines co-expressing T7 RNA polymerase (T7RNAP) and the Tet repressor (TetR) are referred to as wild-type in this study. The conditions for cultivation have been described elsewhere [87,88]. BSF cells used throughout were the single marker strain that constitutively expresses T7RNAP and TetR [21], and were grown in Hirumi modified Iscove’s medium 11 (HMI-11) [89] supplemented with G418 (2.5 μg mL^-1^). BSF were grown at 37°C with 5% (v/v) CO_2_ in humidified atmosphere and kept at cell densities of 1 x 10^5^ to 2 x 10^6^ cells mL^-1^ and diluted with fresh HMI-11 media as required.

For transfections, 10 μg of linearised constructs (see below) were electroporated into 1 x 10^7^ to 2 x 10^7^ cells using an Amaxa Nucleofector 2b device or BTX electroporator, as previously described [87,88]. Stable transformants were selected by clonal dilution in media containing the appropriate selection drugs.

### RNAi constructs

The sequences for all primers used in this study can be found in online supplementary material. RNAi constructs were prepared by amplifying fragments of *Tb*CIA2A, *Tb*CIA2B, and *Tb*MMS19 flanked by *Bam*HI and *Xho*I restriction sites and cloning into the p2T7-177 RNAi vectors [90]. *Tb*MMS19 and *Tb*CIA2A RNAi in PCF were obtained by Gibson assembly using the pTrypSon vector [91]. Double RNAi constructs were generated by ligating a second gene fragment in previously generated single RNAi constructs upon digestion with *Bam*HI and *Spe*I as described before [25]. Constructs were linearized with *Not*I to allow integration into the silent 177 repeats of the *T*. *brucei* minichromosome prior to transfection into PCF 29–13, SmOx or BSF single marker cells. Selection was carried out with 1.25 to 5 μg mL^-1^ phleomycin, or 4 μg mL^-1^ hygromycin B (for the BSF *Tb*MMS19 RNAi cell line).

### Constructs for epitope tagging

C-terminal *in situ* V5 tagging of the *Tb*CIA proteins was performed as described [92]. PCR tagging was performed using a modified version of the pPOTv4 vector in which eYFP was replaced by the sequence of a triple V5 tag. PCR products were electroporated into PCF SmOxP927 cell line and selection was performed with 50 μg mL^-1^ hygromycin B. For C-terminal HA- or PTP-tagging, ~400–1,000 bp upstream of the termination codon of the genes of interest were inserted into the vectors pC-HA-BLA [93] or pC-PTP-PURO [94] using the *Kpn*I and *Afl*II restriction sites. N-terminal PTP-tagging constructs were generated by ligating pN-PTP-PURO [94] with ~400–1,200 bp downstream of the start codon of the respective CIA gene using *Not*I and *Kpn*I restriction sites. The resulting plasmids were linearised at restriction sites within the inserts and transfected into PCF 29–13 or BSF single marker cells then selected with 20 μg mL^-1^ blasticidin or 2 μg mL^-1^ puromycin. The vector for conditional ectopic overexpression of N-terminally tagged proteins was constructed by amplifying the PTP-tag sequence from pN-PTP-PURO and ligating into to the plasmid pLEW82v4 [21] using 5’ *Pac*I and 3’ *Hind*III restriction sites and adding a *Kpn*I recognition sequence downstream of *Pac*I to allow the introduction of the *TbMMS19* ORF or sequences corresponding to its N- or C-terminal domain in frame with the PTP tag. pLEW82-PTP constructs were linearised with *Not*I for integration at the *rRNA* locus, transfected into *Tb*CIA2B-HA PCF cells and selected with 5 μg mL^-1^ puromycin.

### Western blot analyses

Proteins were resolved by SDS-PAGE, transferred to PVDF or nitrocellulose membranes and blocked in phosphate-buffered saline (PBS) with 5% milk for 1 hr at room temperature (RT). Blots were incubated with primary antibodies (see below) overnight at 4 ^o^C, washed three times in PBS-T (PBS supplemented with 0.1% Tween 20), and incubated with the corresponding secondary antibodies for 1 hr at RT before further washes in PBS-T. Fluorescent signals were captured using the Odyssey CLx digital Imaging system (Li-Cor Biosciences) or chemiluminescent signals developed using the Clarity ECL substrate (BioRad). Data for semi-quantitative Western blots were obtained by densitometry using the FIJI package for ImageJ [95]. The following primary antibodies were used in this study: mouse monoclonal α-V5 (1:1.000; Invitrogen), α-tubulin (1:10000), α-TAO (1:100) [31], and α-Strep-Tag (1:500; IBA Life Sciences), mouse polyclonal α-*Tb*PLA1 (1:1000) [30], rabbit polyclonal α-*Tb*ENO (1:2000; a gift from Paul A.M. Michels), α-mtHSP70 (1:1000), α-protein A (PAP antibody; 1:500; Sigma-Aldrich), and rat monoclonal 3F10 α-HA (1:1000; Sigma-Aldrich). In some experiments *Tb*CIA2A and *Tb*CIA2B were detected with specific antibodies (both at 1:200) raised in rabbit using protocols described elsewhere [70–73]. Fluorescent secondary antibodies were goat IgG α-rabbit, α-mouse, or α-rat conjugated to IRDye680 or IRDye800 (Li-Cor Biosciences). HRP-conjugated reagents were from Sigma-Aldrich.

### Confocal imaging

Preparation of C-terminal *in situ*-tagged *Tb*CIA1-V5, *Tb*CIA2A-V5, *Tc*CIA2B-V5 and *Tb*MMS19-V5 for confocal imaging was performed as described elsewhere, with minor modifications [88]. Cells were fixed with 4% (w/v) paraformaldehyde in phosphate buffered saline (PBS), permeabilised with 0.2% (v/v) Triton X-100 in PBS on microscopy slides and then probed with primary antibodies in PBS/gelatin. Monoclonal α-V5 (Life Technologies) and polyclonal anti-*Tb*ENO antibodies were used at 1:1000 and 1:2000 dilution, respectively. As secondary antibodies, Alexa Fluor 488 anti-mouse and Alexa Fluor 555 anti-rabbit (Life Technologies) were used. DNA was visualized using ProLong Gold antifade reagent with DAPI (Life Technologies). Confocal microscopy was performed using an inverted IX81 motorized FluoView FV1000 confocal (Olympus) microscope and detection was carried out with FV1000 software (Olympus). Image analysis was performed using Magic Montage plugin for ImageJ [96] and FIJI [95].

### Homology modelling of protein structures

For generation of structural homology models, amino acid sequences of proteins were submitted to Protein Homology/analogy Recognition Engine v. 2.0 (Phyre2) [53], available at http://www.sbg.bio.ic.ac.uk/phyre2/, using either the normal or intensive modelling modes. The resulting PDB files with the 3D structure of proteins were visualised with MacPyMOL (Schrodinger).

### *In silico* protein-protein docking and interaction hot-spots

PDB files with the 3D structures of proteins were used as input to the ClusPro docking server [59,97], available at https://cluspro.bu.edu/home.php. *Tb*MMS19 was defined as the receptor and *Tb*CIA2B as ligand and all settings were kept as default. Output PBD files containing the top highest scoring models according to the balanced method were downloaded and visualised with MacPyMOL (Schrodinger). PDB files with protein complexes were uploaded to the PredHS server [61], available at http://www.predhs.org/. The predicted interaction hot-spots on the surface of proteins were identified by the SVM algorithm and superimposed on the 3D structure of the complex using MacPyMOL (Schrödinger).

### Recombinant protein expression and purification

PCR amplified sequences corresponding to the N- or C-terminal domains of *Tb*MMS19, or fragments of the latter were cloned into using pGEX-6P-1 (GE Healthcare), using the *Bam*HI and *Not*I restriction sites. The sequence for a Strep-Tag II was included in the antisense primers to generate a C-terminal Strep-Tag II fusion in addition to the N-terminal GST tag encoded in the expression vector. *TbCIA2B* was cloned into pASK-IBA7plus (IBA Life Sciences) using *Eco*RI and *Eco*RV restriction sites and the sequence for a hexahistidine tag was included in the antisense primer to generate a C-terminal 6XHIS fusion in addition to the N-terminal Strep-Tag II present in the vector. Recombinant proteins were expressed in C43 (DE3) pLysS *E*. *coli* [98] carrying the pRARE plasmid for rare codons, grown in terrific broth. r*Tb*CIA2B was purified by immobilised metal affinity chromatography with Ni-NTA agarose (Qiagen) and eluted in EB1 (50 mM Tris.HCl, pH 9, 250 mM NaCl, 0.1% Triton X-100, 1 mM EDTA, 1 mM DTT, 400 mM imidazole, 10% glycerol). Fragments and domains of *Tb*MMS19 were batch purified with Glutathione Sepharose 4B beads (GE Healthcare), followed by Strep-Tactin (IBA Life Sciences) affinity purification and eluted in EB2 (50 mM Tris.HCl, pH 9, 250 mM NaCl, 1% Triton X-100, 0.5% sarkosyl, 1 mM EDTA, 1 mM DTT, 5 mM desthiobiotin, and 10% [v/v] glycerol).

### TAP/MS

Tandem affinity purifications were performed following a standard protocol as described [99], with minor modifications. Briefly, 2.5 litres of PCF expressing PTP tagged proteins were grown to late log phase, centrifuged and washed in ice-cold PBS. Cell pellets were suspended in TLB buffer (20 mM Hepes KOH pH 7.7, 150 mM potassium glutamate, 150 mM sucrose, 3 mM MgCl_2_, 2 mM DTT, 1% [v/v] Triton X-100, Roche cOmplete EDTA-free protease inhibitor cocktail) and lysed on ice with a Dounce homogenizer. Lysates were cleared by centrifugation, filtered into a 10 mL Poly-Prep column (Bio-Rad) and incubated with pre-equilibrated IgG Sepharose 6 Fast Flow resin (GE Healthcare). The resin was washed with PA-150, equilibrated with TEV buffer and incubated overnight with 400U of AcTEV protease (Invitrogen). TEV eluates were collected, added to buffer PC-150 supplemented with 1 mM CaCl_2_ and protease inhibitors, then bound to a pre-equilibrated Anti-Protein C affinity matrix (Sigma-Aldrich) in another Poly-Prep. After extensive washes, proteins were eluted in 1.8 mL of EDTA/EGTA buffer and concentrated with StrataClean resin (Agilent). The resin was pelleted, resuspended in NuPAGE LDS sample buffer (Invitrogen), boiled at 95°C for 10 minutes, and the proteins were resolved in NuPage 4–12% Bis-Tris gels (Invitrogen) before staining with SYPRO Ruby (Molecular Probes). Images were captured in a Typhoon FLA 7000 laser scanner (GE Healthcare). Trypsin digests of excised gel sections were analysed by LC/MS in an ABSciex TripleTOF 5600+ mass spectrometer and the spectra were searched against a *T*. *brucei* protein database [46] using MASCOT. Proteins hits with less than 2 unique peptides were disregarded.

### Co-immunoprecipitation

PCF parasites were washed with ice cold PBS, resuspended in TLB buffer and quickly disrupted with glass beads in a FastPrep machine (MP Biomedicals). Lysates were cleared by centrifugation (16,000 g, 30 minutes, 4°C) and transferred to 1.5 mL tubes containing 25 μL of pre-equilibrated IgG Sepharose 6 Fast Flow resin (GE Healthcare) (for assays with double-tagged cell lines) or 200 pmoles of *Tb*MMS19 GST-fusion proteins immobilised to 25 μL of Glutathione Sepharose 4B, and incubated with rotation for two hours at 4°C. Alternatively, immobilised proteins were incubated with 400 pmoles of purified r*Tb*CIA2B. The resins were washed 4 times with 1 mL of TLB, resuspended in 25 μL of 2 X SDS-PAGE sample buffer and subsequently boiled at 95°C for 10 min. For Strep Tag pull-downs, 400 pmoles of r*Tb*CIA2B were immobilised in 25 μL of Strep-Tactin resin and everything else performed as described above. Interactions were analysed by Western blot after SDS-PAGE.

For V5 co-IP/MS, pellets of 3 x 10^9^ PCF or BSF cells were suspended in PBS, snap-frozen in liquid nitrogen and grinded using a CryoGrinder (OPS Diagnostic) [51]. The cell powder was suspended in 500 μL of lysis buffer (20 mM HEPES, pH 7.4, 150 mM Na-Citrate, 1 mM MgCl_2_, 0.2 mM CaCl_2_, 0.1% [v/v] Triton X-100, and Roche cOmplete EDTA-free protease inhibitor cocktail). Cleared lysates were added to 12 μL of DynaBeads pre-cross-linked with anti-V5 antibody and incubated for two hours at 4°C. Beads were further washed with lysis buffer and proteins were eluted in 100 μL of elution buffer (25 mM Tris.HCl, pH 7.5, 2% [v/v] SDS) at 72°C for 10 minutes. Proteins were precipitated with ethanol, resolved by SDS-PAGE, visualised by silver staining and analysed by Western blot or mass spectrometry.

### Selective permeabilization with digitonin

Cell fractionation using a digitonin gradient was performed as described elsewhere [100]. For co-localisation, 1 x 10^7^ cells were suspended in 100 μL of FB (20 mM Tris-HCl, 0.6 M sorbitol, 1 mM DTT, Roche cOmplete protease inhibitor cocktail; pH 7.5) containing concentrations of 0.01 to 1 mg mL^-1^ of digitonin. Cells were incubated on ice for 5 min and centrifuged at 16,000 g for 5 min at 4°C. The supernatant was transferred to a clean 1.5 mL tube and evaporated in a SpeedVac until almost dry. The pellet was suspended in 20 μL of 2 X SDS-PAGE sample buffer, boiled for 10 min at 95°C and resolved by SDS-PAGE. Protein release in each fraction was detected by a semi-quantitative Western blot. Cytosolic and organellar fractions for other assays were prepared by suspending 1 x 10^8^ cells in 1 mL FB containing 0.15 mg mL^-1^ digitonin. The soluble supernatant was considered the cytosolic fraction. The pellet was washed once with 1 mL of FB, incubated for 10 min on ice with 1 mL FB with 0.5% (v/v) Triton X-100 and centrifuged at 16,000 g for 5 min at 4°C. The resulting supernatant was considered the organellar fraction.

### Crude cell fractionation analysis

The cytosol was separated from the organellar fraction as described elsewhere [101]. Mid-log cells expressing the V5-tagged proteins were harvested at 1000 g for 10 min at 4°C, washed with ice cold SHE buffer (25 mM HEPES, pH 7.4, 250 mM sucrose, 1 mM EDTA), resuspended in fresh SHE buffer to a final concentration of 5 x 10^9^ cells mL^-1^, and the protein concentration was determined according to Bradford. One milligram protein aliquots were suspended in Hanks’ balanced salt solution (HBSS) (1.26 mM CaCl_2_, 5.33 mM KCl, 0.44 mM KH_2_PO_4_, 0.81 mM MgSO_4_, 138 mM NaCl, 4 mM NaHCO_3_, 0.3 mM Na_2_HPO_4_, 5.6 mM glucose, pH 7.3) and digitonin was added to the final concentration of 0.4 μg μL^-1^. After vortexing, the suspension was incubated at RT for 5 min, and followed by centrifugation at 14000 g at RT for 2 min. The supernatant represented the cytosolic fraction, while the pellet was washed once with HBSS and then resuspended in HBSS containing 0.1% (v/v) Triton X-100 and incubated on ice for 5 min. After centrifugation, the supernatant was collected as the organellar fraction. The pellet was washed once more with HBSS and then resuspended in a volume equal to the previous two fractions and analyzed by Western blotting. This final pellet fraction contains proteins that are insoluble or strongly associated to membranes.

### Aconitase activity measurement

Aconitase activity was measured as previously described by monitoring the increase of the absorbance at 240 nm due to the conversion of isocitrate into *cis*-aconitate [102]. Two hundred microliters of lysates were added to 1.3 mL of aconitase buffer (50 mM Tris.HCl, 1 mM DTT, 20 mM DL-isocitric acid or sodium citrate; pH 7.4) and incubated at 25°C. The rate of increase of the absorbance at 240 nm per min (ΔA_240 nm_/min) was monitored for 30 min in a Varian Cary 50 UV/Vis spectrophotometer. A blank reaction without cell lysate was run in parallel. Specific activity was obtained by dividing the measured aconitase activity (mU mL^-1^) by protein concentration in the sample. Uninduced controls were considered as 100% of activity.

### Measurement of protein-bound iron

Cellular fractions from digitonin fractionation were concentrated in a SpeedVac (Thermo) and the iron content measured by the Ferene method, as described by [103],. The pellets were thoroughly suspended in 100 μL of milliQ water, mixed with 100 μL of 1% HCl, incubated for 10 min at 100°C, quickly cooled down on ice and centrifuged (12,000 g, 5 min). Subsequently, 500 μL of 7.5% ammonium acetate, 100 μL 4% ascorbic acid and 100 μL of 2.5% SDS were added to the samples and vortexed. The samples were centrifuged again (12,000 g, 10 min) and 855 μL of the supernatant was transferred to a semi-micro cuvette to which 95 μL of 6.2 mM Ferene (Sigma) were added. The absorbance of the ferrous-ferene complex at 593 nm was corrected for turbidity by subtraction of the absorbance at 800 nm and measured in a Varian Cary 50 UV/Vis spectrophotometer. Iron content was estimated by interpolation from a standard curve of ferrous sulphate (2,000–12.5 ng) using least squares linear regression.

### Resazurin cell viability assay

The Alamar Blue assay was used to assess viability of cells exposed to DNA damaging agents or DFO. In this assay, the resazurin salt is reduced to resorufin, which emits a fluorescent signal proportional to the number of viable cells [104]. Cell densities of exponentially growing cells were adjusted to 1 x 10^6^ or 5 x 10^4^ cells mL^-1^ for PCF and BSF trypanosomes, respectively, to generate a 2x working cell suspension. One hundred microliters of cell suspension were added in quadruplicate to 96-well plates containing 100 μL per well of 2-fold serial dilutions of drugs. Wells without drugs or without cells served as maximum growth control and blank, respectively. PCF cells were grown for 48 hrs at 28°C, while BSF were incubated for 72 hrs at 37°C, after which 10 μL of a 1.1 mg mL^-1^ solution of resazurin (Sigma) were dispensed to each well and the plates were incubated for another 6 hrs. The fluorescent signal was measured in a FLx800TM Microplate reader (BioTek) with excitation wavelength set at λ_530_ and emission at λ_590_. All EC_50_s (concentration of a compound that reduces cell growth by 50%) were calculated by nonlinear regression using the software Prism 7.0 (GraphPad Inc.). DFX, methyl methane sulfonate (MMS), 4-nitroquinoline 1-oxide (4NQO), hydroxyurea, and camptothecin were purchased from Sigma-Aldrich, and phleomycin (Zeocin) was purchased from Thermo Fisher.

### Yeast complementation

Complementation experiments were carried out in *Saccharomyces cerevisiae* strain W303-1A as WT (*MATa, ura3-1, ade2-1, trp1-1, his3-11,15, leu2-3,112*). The galactose-regulatable mutants used were *GalL-MMS19* and *Gal-CIA2* [14,43]. The latter mutant strain was constructed by homologous recombination in which the upstream promoter region of *CIA2* was replaced by a PCR product containing the NatNT2 resistance marker gene and the GAL promoter. PCR analysis of chromosomal DNA confirmed correct insertion of the promoter. Yeast cells were grown in minimal (SC) media, containing galactose or glucose at a concentration of 2% (m/v) [105]. The yeast MMS19-encoding gene was cloned into the *Sma*I and *Xho*I sites of the pRS424-TDH3 vector [106]. For control of the rescue by yeast Cia2, the Cia2-encoding sequence with 500 bp natural promoter (NP) sequence was amplified from yeast DNA and cloned into the *Sac*I and *Xho*I sites of pRS416-MET25. *Tb*CIA2A and *Tb*CIA2B genes were amplified from *T*. *brucei* DNA and cloned into the *BamH*I and *Sal*I sites of pRS416-MET25. *Tb*MMS19 (Tb927.8.3920) was cloned into pRS424-TDH3 in two steps. *Spe*I-*BamH*I and *BamH*I-*Cla*I fragments were consecutively PCR-amplified and cloned. The *BamH*I site, which is lacking in the *Tb*MMS19 gene, introduces silent mutations at amino acids 514–515 (Gly-Ser). After transformation of plasmids into *GalL-MMS19* or *Gal-CIA2* cells, growth in liquid minimal media supplemented with 2% galactose was carried out for 16 h. Then cells were shifted to the same medium, but with 2% glucose for 16 h (*Gal-CIA2*) or 16 and 24 h (*GalL-MMS19*). Cell suspensions were diluted to an optical density of 0.5 at 600 nm and 5 μl aliquots, including four consecutive 10-fold serial dilutions, were spotted on agar plates. Plates containing minimal media supplemented with 2% galactose or glucose were incubated at 30°C for 48 h and photographed.

### *In vivo* infectivity

Mice had food and fresh water *ad libitum*. The experiment was approved by our institution’s Animal Ethics Committee. To determine the infectivity of trypanosomes depleted for *Tb*CIA2B or *Tb*MMS19, six groups of BALB/C mice (uninduced and RNAi-induced *Tb*CIA2B, uninduced and RNAi-induced *Tb*MMS19, wild type single marker [WT SM] cells with and without doxycycline). Each group consisted of 5 females (8 to 9 weeks old) which were infected intraperitoneally with 10,000 BSF cells. In their drinking water, the induced groups received 1 mg/ml doxycycline sweetened with 50 mg/ml sucrose, starting 2 days before the infection. The survival was recorded twice a day. Survival data was plotted using Prism 7.

## Supporting information

S1 Fig Double knockdowns of the CIA targeting complex cause a defect in cell growth in both PCF and BSF trypanosomes. Growth curves of double RNAi cells lines for *Tb*CIA1-*Tb*CIA2B and *Tb*CIA2A-*Tb*CIA2B in PCF (A and B) and BSF (C and D) cells were grown in presence (Tet+) and absence (Tet-) of tetracycline for 10 and 8 days, respectively.
(TIF)

S2 Fig Survival curves upon infection with CTC RNAi cell lines. Survival of mice infected with BSF *Tb*MMS19 (A) and *Tb*CIA2B (B) RNAi cell lines, uninduced (-) and induced (+) with doxycycline. Wild type (SM) was used as controls, also in the absence (-) and presence (+) of doxycycline. Five mice per group were used. Induced (+) cell lines are nudged in the graph for easier visualisation of the overlapping curves.
(TIF)

S3 Fig Homology model of the *T*bMMS19 tertiary structure and HEAT repeat detection. (A) Primary sequence of *Tb*MMS19 with the centres of the HEAT repeats highlighted in a scale of red according to the probability of the respective residue to be in the centre of a HEAT repeat unit, as calculated by Ard2 [55].(B) Predicted 3D structure of *Tb*MMS19 with HEAT repeats highlighted in red. The homology model for *Tb*MMS19 was created using Phyre2 [53].(C) The N-and C-terminal domains of *Tb*MMS19 are represented in blue and red, respectively. The boundaries of the domains were defined by alignment with human MMS19. Letters A-C represent functional domains of the human protein. Grey boxes correspond to HEAT repeats annotated in the Uniprot database for MMS19 (accession number Q9T76), or identified as described in (B) for *Tb*MMS19.
(PNG)

S4 Fig **N- and C-termini of *Tb*MMS19 interact with *Tb*CIA1**. Tandem affinity purifications of PTP-TbMMS19 (3), PTP-TbMMS19-NTD (4), and PTP-TbMMS19-CTD (5), mock (1) and (2) empty plasmid, observed in a SYPRO Ruby-stained SDS-PAGE gel.
(PNG)

S5 Fig Additional models for the complex *Tb*MMS19-*Tb*CIA2B. Three-dimensional models for the proteins were created using Phyre2 [53] and the best predictions were used for docking with ClusPro [59]. The five highest-scoring complexes (balanced score), were analysed with PredHS [61] to identify the key residues for interaction with *Tb*CIA2B at the binding surface of *Tb*MMS19. *Tb*CIA2B is depicted as blue mesh in (A) and the N and C-terminal domains of *Tb*MMS19 are shown in (A) and (B) as grey and green surfaces, respectively. *Tb*CIA2B was omitted in (B) to uncover the residues at the contact surface, which are shown in a scale of red according to their associated SVM hot-spot score. Except in model 5, the residues more likely to be hot-spots of interaction are predicted to be in the C-terminal domain of *Tb*MMS19. MacPyMOL (Schrödinger, LLC) was used to generate the figures based on the output of PredHS, ClusPro and Phyre2.
(PNG)

S1 Table DNA damage induced by genotoxic drugs. (DOCX)

S2 Table Mass spectrometry data for the identified CTC members in TAP/PTP. (DOCX)

S3 Table Mass spectrometry data for the identified CTC members in V5-CoIP in PCF trypanosomes. (DOCX)

S4 Table Mass spectrometry data for the identified CTC members in V5-CoIP in BSF trypanosomes. (DOCX)

S5 Table Mass spectrometry data for PTP-tagged and co-immunoprecipitated V5-tagged CTC components (complete data). (XLSX)
