# Supplementary figures and images for: Branched late-steps of the cytosolic iron-sulphur cluster assembly machinery of Trypanosoma brucei
Source: PLoS Pathog. 2018 Oct 22;14(10):e1007326. doi: 10.1371/journal.ppat.1007326 (PMC6211773; doi:10.1371/journal.ppat.1007326)

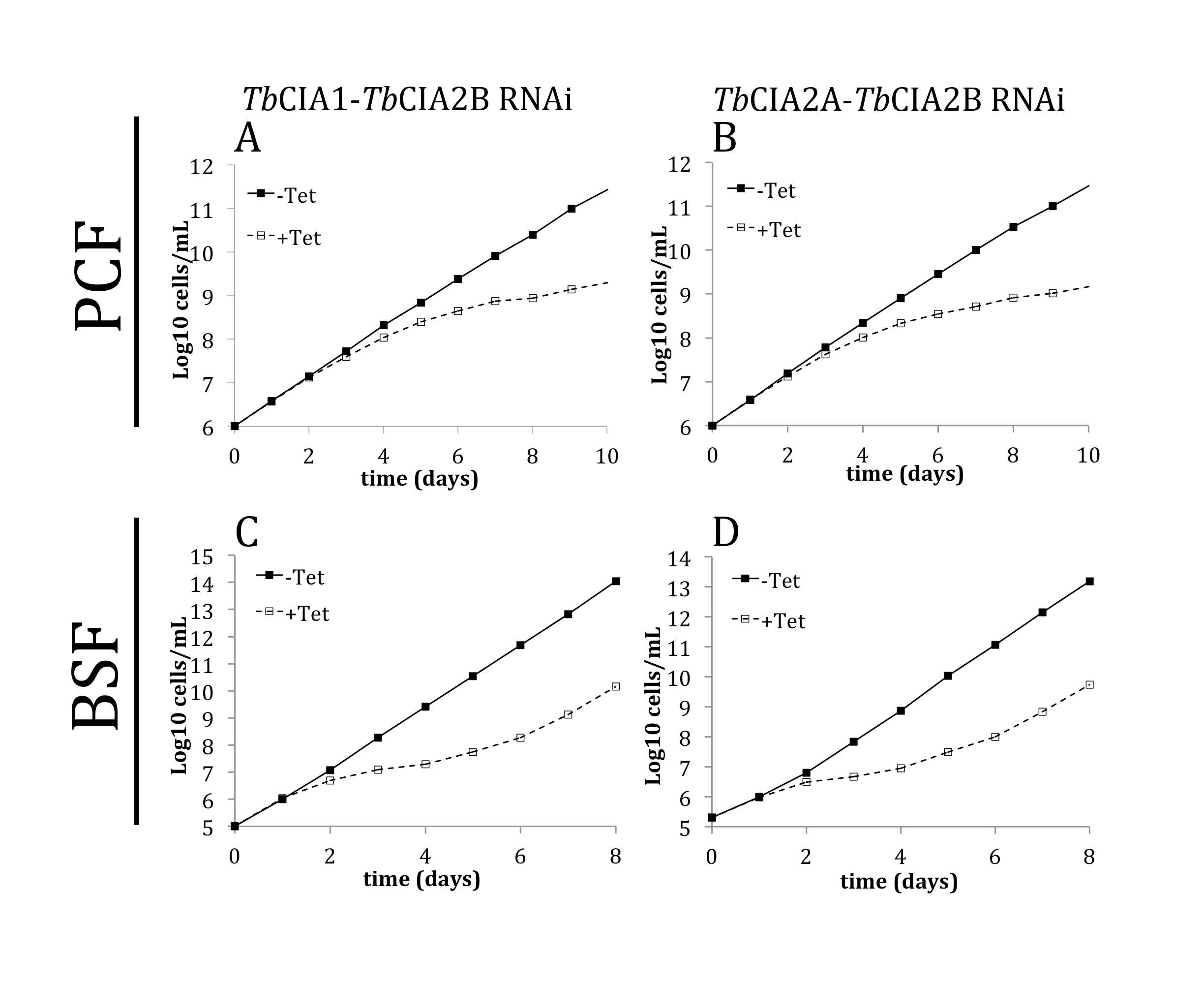

Supplement: S1 Fig — Growth curves of double RNAi cells lines for TbCIA1-TbCIA2B and TbCIA2A-TbCIA2B in PCF (A and B) and BSF (C and D) cells were grown in presence (Tet+) and absence (Tet-) of tetracycline for 10 and 8 days, respectively. (TIF) [file ppat.1007326.s001.tif]

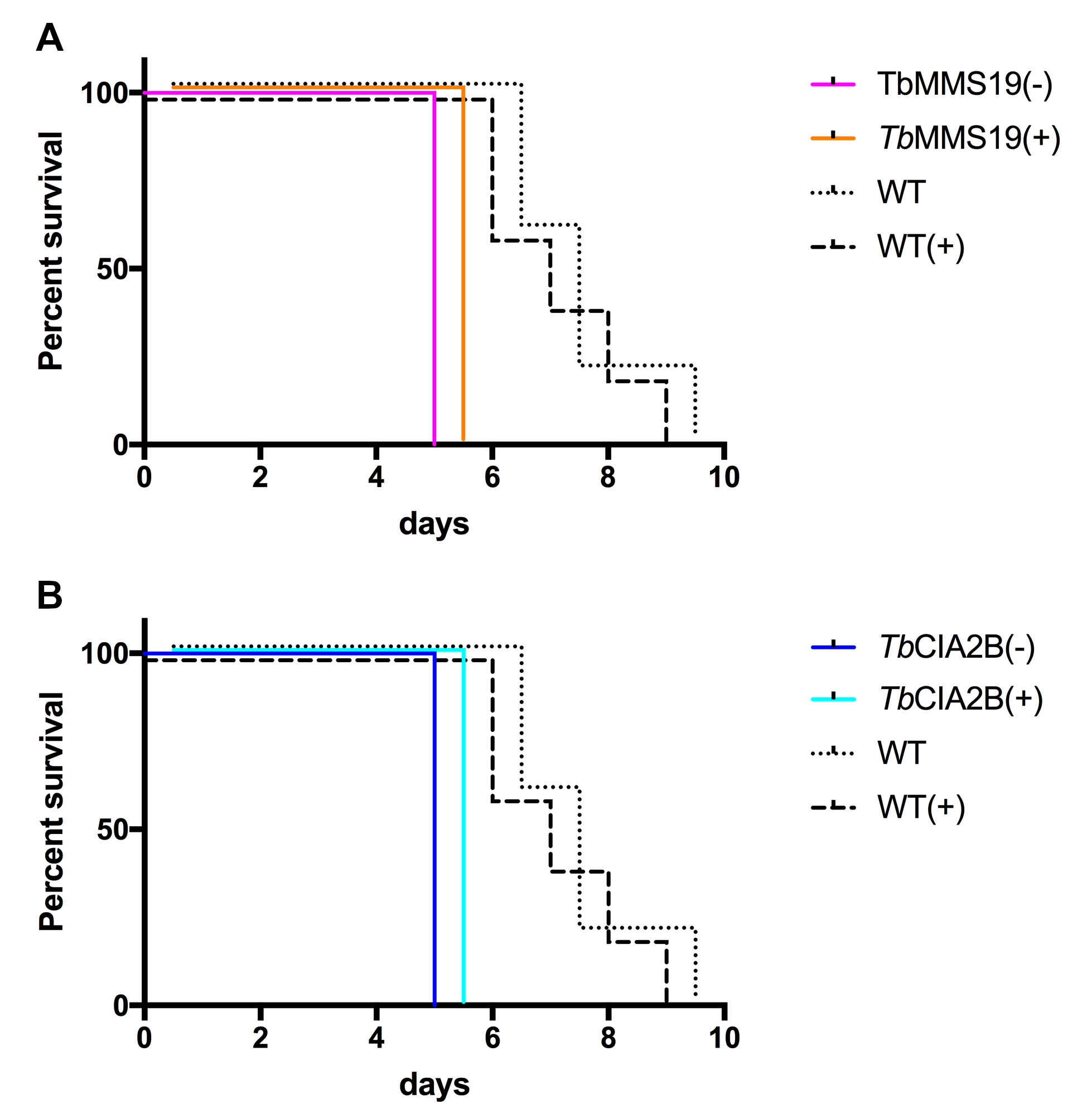

Supplement: S2 Fig — Survival of mice infected with BSF TbMMS19 (A) and TbCIA2B (B) RNAi cell lines, uninduced (-) and induced (+) with doxycycline. Wild type (SM) was used as controls, also in the absence (-) and presence (+) of doxycycline. Five mice per group were used. Induced (+) cell lines are nudged in the graph for easier visualisation of the overlapping curves. (TIF) [file ppat.1007326.s002.tif]

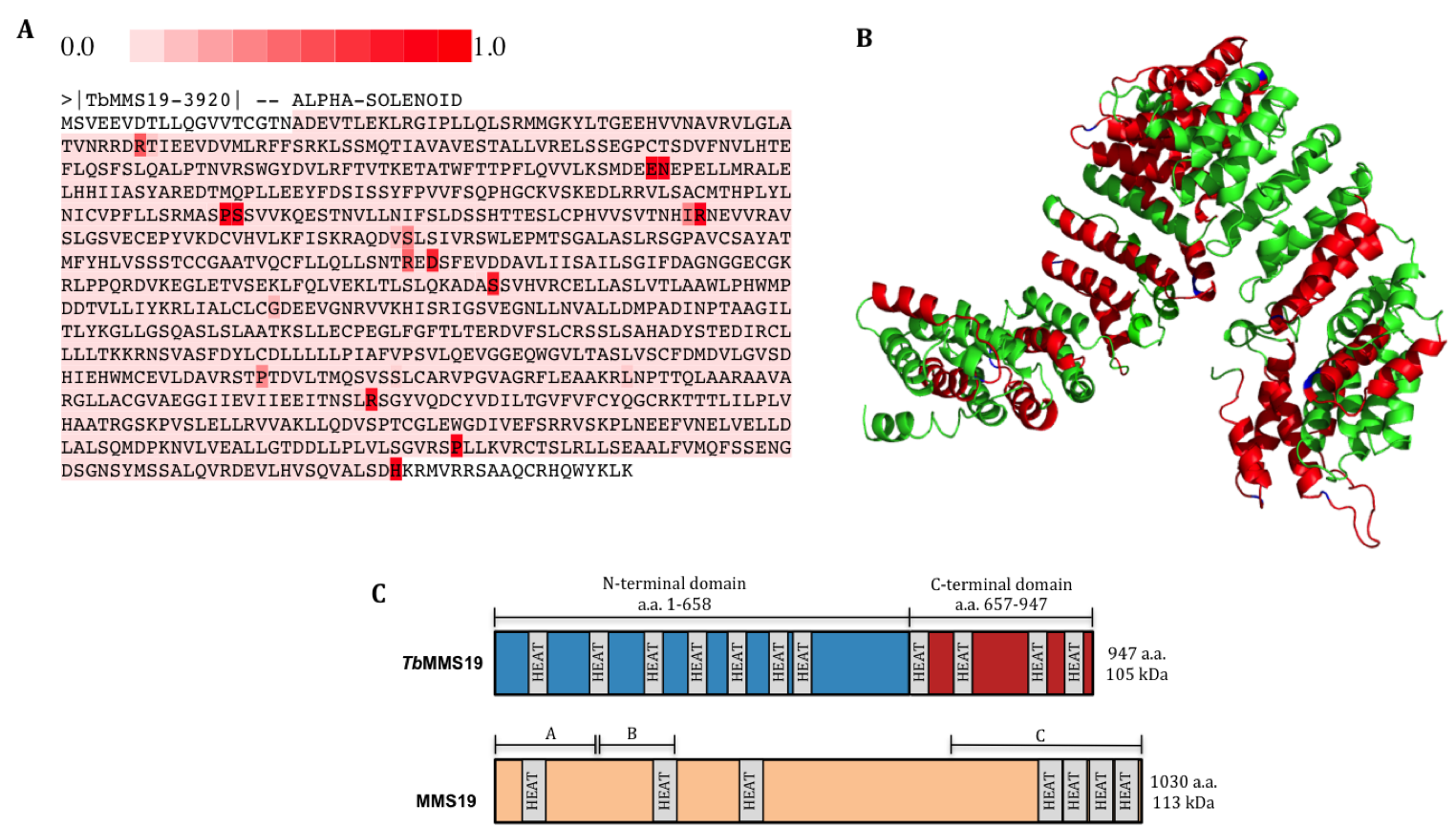

Supplement: S3 Fig — (A) Primary sequence of TbMMS19 with the centres of the HEAT repeats highlighted in a scale of red according to the probability of the respective residue to be in the centre of a HEAT repeat unit, as calculated by Ard2 [55].(B) Predicted 3D structure of TbMMS19 with HEAT repeats highlighted in red. The homology model for TbMMS19 was created using Phyre2 [53].(C) The N-and C-terminal domains of TbMMS19 are represented in blue and red, respectively. The boundaries of the domains were defined by alignment with human MMS19. Letters A-C represent functional domains of the human protein. Grey boxes correspond to HEAT repeats annotated in the Uniprot database for MMS19 (accession number Q9T76), or identified as described in (B) for TbMMS19. (PNG) [file ppat.1007326.s003.png]

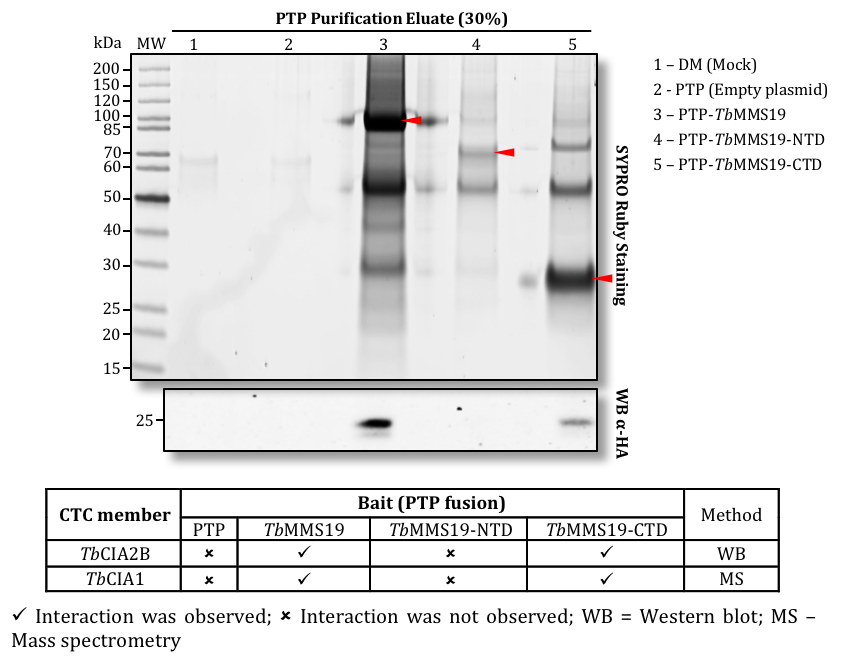

Supplement: S4 Fig — N- and C-termini of TbMMS19 interact with TbCIA1. Tandem affinity purifications of PTP-TbMMS19 (3), PTP-TbMMS19-NTD (4), and PTP-TbMMS19-CTD (5), mock (1) and (2) empty plasmid, observed in a SYPRO Ruby-stained SDS-PAGE gel. (PNG) [file ppat.1007326.s004.png]

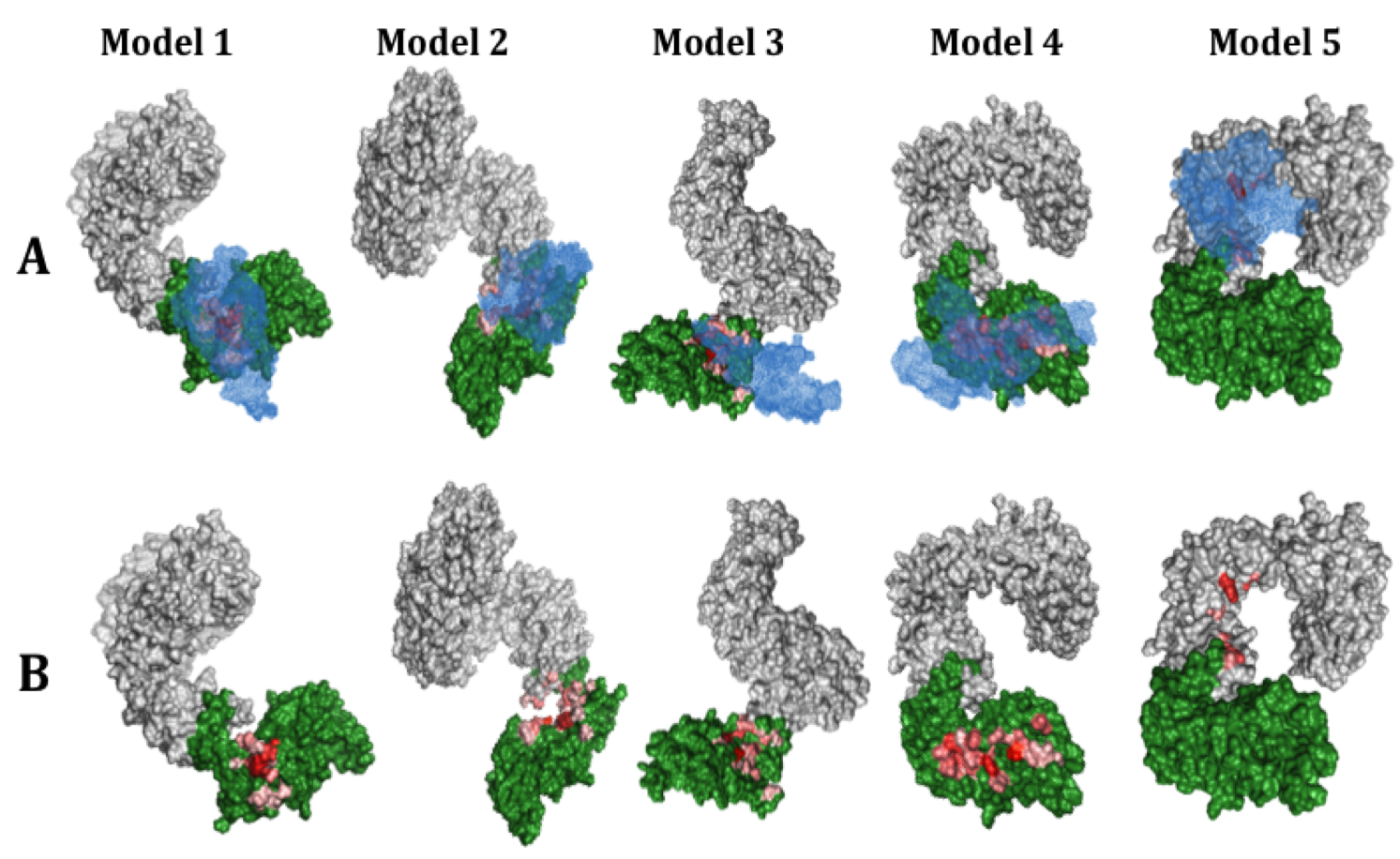

Supplement: S5 Fig — Three-dimensional models for the proteins were created using Phyre2 [53] and the best predictions were used for docking with ClusPro [59]. The five highest-scoring complexes (balanced score), were analysed with PredHS [61] to identify the key residues for interaction with TbCIA2B at the binding surface of TbMMS19. TbCIA2B is depicted as blue mesh in (A) and the N and C-terminal domains of TbMMS19 are shown in (A) and (B) as grey and green surfaces, respectively. TbCIA2B was omitted in (B) to uncover the residues at the contact surface, which are shown in a scale of red according to their associated SVM hot-spot score. Except in model 5, the residues more likely to be hot-spots of interaction are predicted to be in the C-terminal domain of TbMMS19. MacPyMOL (Schrödinger, LLC) was used to generate the figures based on the output of PredHS, ClusPro and Phyre2. (PNG) [file ppat.1007326.s005.png]
