# Supplementary material for: Branched late-steps of the cytosolic iron-sulphur cluster assembly machinery of Trypanosoma brucei
Source: PLoS Pathog. 2018 Oct 22;14(10):e1007326. doi: 10.1371/journal.ppat.1007326 (PMC6211773; doi:10.1371/journal.ppat.1007326)
Supplement: S1 Table — (DOCX) [file ppat.1007326.s006.docx]

**Table S1: DNA damage induced by genotoxic drugs**

| *Tb*CIA2B | | | | | | |
| --- | --- | --- | --- | --- | --- | --- |
| Drug | EC_50_ ± SEM (µM) | | | | | |
|  | PCF | | *p* value summary | BSF | | *p* value summary |
|  | Tet - | Tet + |  | Tet - | Tet + |  |
| MMS | 51.1±7.2 | 75.9±10.5 | ns | 53.2±0.4 | 51.3±6.4 | ns |
| Hydroxyurea | 267.9±36.4 | 593.3±92.2 | * | 35.9±0.6 | 40.8±0.4 | ns |
| Camptothecin | 2±0.2 | 5.0±0.5 | ** | 0.7±0.1 | 0.7±0.1 | ns |
| 4NQO | 0.30±0.03 | 0.23±0.02 | ns | - | - | - |
| Phleomycin | - | - | - | 0.29±0.03 | 0.25±0.01 | ns |
| *Tb*MMS19 | | | | | | |
| Drug | EC_50_ ± SEM (µM) | | | | | |
|  | PCF | | *p* value summary | BSF | | *p* value summary |
|  | Tet - | Tet + |  | Tet - | Tet + |  |
| MMS | 63±10.4 | 48.8±7.1 | ns | 48.9±3.7 | 46.9±7.1 | ns |
| Hydroxyurea | 310.2±7.1 | 291.8±51.3 | ns | 34.4±2.3 | 34.1±1 | ns |
| Camptothecin | 1.3±0.2 | 1.3±0.06 | ns | 0.7±0.1 | 0.7±0.1 | ns |
| 4NQO | 0.26±0.04 | 0.21±0.05 | ns | - | - | - |
| Phleomycin | - | - | - | 0.33±0.08 | 0.32±0.08 | ns |

ns = non-significant; * p< 0.05; ** p<0.01 (two tailed paired t test). MMS= Methyl methanesulfonate;

4NQO= 4-Nitroquinoline 1-oxide
