# Supplementary material for: Branched late-steps of the cytosolic iron-sulphur cluster assembly machinery of Trypanosoma brucei
Source: PLoS Pathog. 2018 Oct 22;14(10):e1007326. doi: 10.1371/journal.ppat.1007326 (PMC6211773; doi:10.1371/journal.ppat.1007326)
Supplement: S2 Table — (DOCX) [file ppat.1007326.s007.docx]

**Table S2: Mass spectrometry data for the CTC members identified in PTP (TAP) pull-downs in PCF parasites**

| **Bait**  **(PTP fusion)** | **Identified CTC members**  **Number of distinct sequences / Sequence coverage (%)** | | | |
| --- | --- | --- | --- | --- |
|  | ***Tb*CIA2B** | ***Tb*Cia1** | ***Tb*MMS19** | ***Tb*CIA2A** |
| Mock | 0 | 0 | 0 | 0 |
| *Tb*CIA2B | 5 (42) | 2 (6) | 31 (41) | 0 |
| *Tb*Cia1 | 11 (76) | 18 (61) | 28 (28) | 3 (20) |
| *Tb*MMS19 | 11 (82) | 12 (39) | 33 (36) | 0 |
