# Supplementary material for: Branched late-steps of the cytosolic iron-sulphur cluster assembly machinery of Trypanosoma brucei
Source: PLoS Pathog. 2018 Oct 22;14(10):e1007326. doi: 10.1371/journal.ppat.1007326 (PMC6211773; doi:10.1371/journal.ppat.1007326)
Supplement: S3 Table — (DOCX) [file ppat.1007326.s008.docx]

**Table S3: Mass spectrometry data for the CTC members identified in anti-V5 pull-downs in PCF parasites.**

| **Bait**  **(V5 fusion)** | **Identified CTC members**  **Number of unique peptides / Number of all peptides** | | | |
| --- | --- | --- | --- | --- |
|  | ***Tb*CIA2B** | ***Tb*Cia1** | ***Tb*MMS19** | ***Tb*CIA2A** |
| Mock | - | - | - | - |
| *Tb*CIA2B | 9 (9) | 19 (19) | 3 (3) | - |
| *Tb*CIA1 | 7 (7) | 29 (29) | 2 (37) | 8 (8) |
| *Tb*MMS19 | 8 (8) | 23 (23) | 43 (43) | - |
| *Tb*Cia2A | - | 22 (22) | - | 14 (14) |

| **Bait**  **(V5 fusion)** | **Identified CTC members**  **Number of unique peptides / Number of all peptides** | |
| --- | --- | --- |
|  | ***Tb*Nar1** |  |
| Mock | - |  |
| *Tb*CIA2B | - |  |
| *Tb*CIA1 | 9 (9) |  |
| *Tb*MMS19 | 7 (7) |  |
| *Tb*Cia2A | 2 (2) |  |
