# Supplementary material for: Branched late-steps of the cytosolic iron-sulphur cluster assembly machinery of Trypanosoma brucei
Source: PLoS Pathog. 2018 Oct 22;14(10):e1007326. doi: 10.1371/journal.ppat.1007326 (PMC6211773; doi:10.1371/journal.ppat.1007326)
Supplement: S4 Table — (DOCX) [file ppat.1007326.s009.docx]

**Table S4: Mass spectrometry data for the CTC members identified in anti-V5 pull-downs from BSF parasites**

| **Bait**  **(V5 fusion)** | **Identified CTC members**  **Number of unique peptides / Number of all peptides** | | | |
| --- | --- | --- | --- | --- |
|  | ***Tb*CIA2B** | ***Tb*Cia1** | ***Tb*MMS19** | ***Tb*CIA2A** |
| Mock | - | 1 (1) | 2 (2) | - |
| *Tb*CIA2B | 5 (5) | 17 (17) | 38 (38) | - |
| *Tb*CIA1 | 5 (5) | 19 (19) | 28 (28) | 5 (5) |
| *Tb*MMS19 | 5 (5) | 15 (15) | 36 (36) | - |
| *Tb*Cia2A | - | 7 (7) | 2 (2) | 9 (9) |
